# Structure and expression of two nuclear receptor genes in marsupials: insights into the evolution of the antisense overlap between the α-thyroid hormone receptor and Rev-erbα

**DOI:** 10.1186/1471-2199-11-97

**Published:** 2010-12-10

**Authors:** Brandon C Rindfleisch, M Scott Brown, John L VandeBerg, Stephen H Munroe

**Affiliations:** 1 Department of Biological Sciences, Marquette University, Milwaukee, WI, USA; 2 Current address: Department of Pharmacology and Toxicology, Medical College of Wisconsin, Milwaukee, WI, USA; 3 Current address: Department of Molecular Genetics and Cell Biology, University of Chicago, Chicago, IL, USA; 4 Department of Genetics and Southwest National Primate Research Center, Southwest Foundation for Biomedical Research, San Antonio, TX, USA

## Abstract

**Background:**

Alternative processing of α-thyroid hormone receptor (TRα, NR1A1) mRNAs gives rise to two functionally antagonistic nuclear receptors: TRα1, the α-type receptor, and TRα2, a non-hormone binding variant that is found only in mammals. TRα2 shares an unusual antisense coding overlap with mRNA for Rev-erbα (NR1D1), another nuclear receptor protein. In this study we examine the structure and expression of these genes in the gray short-tailed opossum, *Monodelphis domestica*, in comparison with that of eutherian mammals and three other marsupial species, *Didelphis virginiana, Potorous tridactylus *and *Macropus eugenii*, in order to understand the evolution and regulatory role of this antisense overlap.

**Results:**

The sequence, expression and genomic organization of mRNAs encoding TRα1 and Rev-erbα are very similar in the opossum and eutherian mammals. However, the sequence corresponding to the TRα2 coding region appears truncated by almost 100 amino acids. While expression of TRα1 and Rev-erbα was readily detected in all tissues of *M. domestica *ages 0 days to 18 weeks, TRα2 mRNA was not detected in any tissue or stage examined. These results contrast with the widespread and abundant expression of TRα2 in rodents and other eutherian mammals. To examine requirements for alternative splicing of TRα mRNAs, a series of chimeric minigenes was constructed. Results show that the opossum TRα2-specific 5' splice site sequence is fully competent for splicing but the sequence homologous to the TRα2 3' splice site is not, even though the marsupial sequences are remarkably similar to core splice site elements in rat.

**Conclusions:**

Our results strongly suggest that the variant nuclear receptor isoform, TRα2, is not expressed in marsupials and that the antisense overlap between TRα and Rev-erbα thus is unique to eutherian mammals. Further investigation of the TRα and Rev-erbα genes in marsupial and eutherian species promises to yield additional insight into the physiological function of TRα2 and the role of the associated antisense overlap with Rev-erbα in regulating expression of these genes.

## Background

Recent genome-wide studies have revealed far more extensive transcription of mammalian genomes than previously recognized [1-6]. One consequence of this pervasive transcription is the occurrence of widespread bidirectional transcription that results in overlapping transcripts antisense to most known mRNAs [5,7-9]. Such antisense transcription takes several forms. Both long and short non-coding antisense transcripts have been described overlapping either the 5' or 3' ends of mRNAs. In other instances two mRNAs encoded on opposite strands share such an overlap. Although regulatory implications of antisense transcription are unclear, many studies suggest that the mechanisms associated with expression of antisense RNA in eukaryotes are remarkably diverse and mechanistically complex [9-14].

One of the first examples of an antisense overlap between two mRNAs involves two nuclear receptor genes, the α-type thyroid hormone receptor gene (TRα; also NR1A1 or THRA) and the Rev-erbα gene (NR1D1) [15-17]. These genes share an antisense overlap that reflects the presence of a novel alternatively spliced mRNA that is highly conserved in most mammals but is absent in non-mammalian vertebrates [18]. Alternative processing of TRα pre-mRNAs yields two functionally distinct proteins. One is the canonical thyroid hormone (T3) receptor, TRα1, that activates target genes in a thyroid hormone (T3)-dependent manner. The other, TRα2, has a novel C-terminal sequence encoded by an exon antisense to Rev-erbα. TRα2 mRNA lacks both the hormone-binding domain and a critical activation domain present in TRα1 and may function as a weak dominant negative repressor, repressing expression of genes activated by TRα1 in the presence of T3 [17]. Rev-erbα has a DNA-binding specificity distinct from TRα1 and TRα2. Like TRα1, Rev-erbα possesses a canonical ligand binding domain but, like TRα2, it lacks the AF-2 activation domain. Thus, both Rev-erbα and TRα2 are constitutive repressors, although they bind distinct sets of target genes.

TRα1 and Rev-erbα play important roles in metabolic and developmental regulation [19-21]. TRα1 mediates T3 activity critical for vertebrate development and homeostatic metabolism. Rev-erbα plays critical roles in adipogenesis and lipid metabolism, while occupying a specific node in the mammalian circadian clock [22-26]. While previously considered a ligandless orphan receptor, Rev-erbα has recently been shown to bind heme specifically within its canonical ligand binding domain (LBD) [24,26]. Interactions of Rev-erbα with its co-repressors are enhanced in the presence of heme and, with heme bound, may respond to physiological regulators such as nitric oxide [23,27,28]. Thus, Rev-erbα has emerged as an important integrator of inputs from multiple physiologic pathways [29-31].

The overlap architecture of the region encoding TRα and Rev-erbα (here referred to as the TRα/Rev-erbα locus) and the physiological importance of these nuclear receptors raise important questions relating to the function, evolution and regulation of this locus. The evolutionary constraints imposed on such a bidirectional coding sequence argue that it is highly unlikely that such an overlap would occur without some functional benefit. Several lines of evidence support the hypothesis that expression of Rev-erbα mRNA may affect the balance between TRα1 and TRα2 through inhibition of TRα2 splicing [32-35]; however, such an interaction has not been demonstrated under physiological conditions.

In this study we first analyze the structure of the TRα/Rev-erbα locus in *Monodelphis domestica*, the gray short-tailed opossum, the first published marsupial genome [36], and compare it with that of other marsupials. Individually, TRα and Rev-erbα genes and transcripts are highly conserved in the opossum in comparison with those of eutherian mammals. However, the region corresponding to the Rev-erbα/TRα2 overlap in eutherian mammals displays several divergent features. Most of the C-terminal coding sequence unique to TRα2 is missing in *M. domestica *due to the introduction of an in-frame stop codon that eliminates the bidirectional coding overlap with Rev-erbα. Furthermore, expression of TRα2 mRNA was not detected in the opossum in any of a variety of tissues or developmental stages. These results are surprising given the remarkable conservation of TRα2 and its abundant and developmentally regulated expression in eutherian mammals. Analysis of chimeric minigenes comprised of opossum and rat sequences demonstrated that opossum sequences homologous to the TRα2-specific splice site (3'ss) are very poorly utilized in comparison with similar rat minigenes. These studies thus document the evolution of a novel alternatively spliced exon in mammals and the creation of antisense overlap between two important regulatory proteins.

## Results

### Structures of eutherian TRα1 and Rev-erbα mRNAs are conserved in marsupials

**Figure 1:**
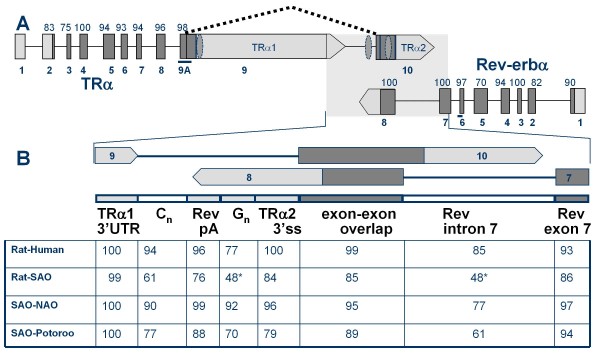
**The TRα/Rev-erbα locus is conserved in marsupials**. **A**. Structure of TRα and Rev-erbα genes is shown schematically for rat and *M. domestica*. Rectangular boxes indicate exons, boxes terminating in arrows represent poly(A) sites, dark shading indicates coding sequences and bold vertical lines the alternative 5'ss splice site for exon 9A and stop codons for TRα1 (exon 9) and TRα2 (exon 10). Alternative splicing of TRα2 (exon 9A to exon 10) is indicated by dotted angle line. Percent amino acid identity is indicated above each exon and exon numbering is shown in bold below. Conservation of TRα2 exon 10 is described in Figure 2. Probes for RNase protection assays are indicated by bold lines under TRα exon 9A and Rev-erbα exon 6. Hatched ellipses indicate positions of elements that enhance TRα2 mRNA splicing [38]. **B**. (top) To-scale enlargement of the antisense overlap showing portions of exons from TRα1, TRα2 and Rev-erbα mRNAs within shaded region in panel A. Shaded horizontal bar indicates eight subregions based on an alignment of eutherian and marsupial sequences across this region (additional file 2). C_n _and G_n _represent regions that include multiple runs of C and G residues, respectively. Rev pA is the conserved region centered on the Rev-erbα poly(A) site. Table at bottom shows percent identity for selected pairwise comparsions: SAO, South American opossum (*M. domestica*); NAO, North American opossum (*D. viriginiana*); potoroo, *P. tridactylus*. Asterisks indicate highly gapped regions in the alignment.

Inspection of the genome of *Monodelphis domestica *[36] reveals a single locus on chromosome 2 in which the TRα and Rev-erbα genes are arrayed in a convergent orientation similar to that in rat and other mammalian genomes (Figure 1). The coding regions for TRα1 (additional file 1 Panel A) and Rev-erbα are separated by only 3.85 kb, similar to the close spacing of the coding regions in the rat genome (3.71 kb). Furthermore, the non-coding sequence extending across and between the 3' ends of these genes is also very well conserved (Figure 1B). The most striking conservation is located at the extreme 3' end of TRα1 mRNA, in which a sequence of 140 bp is almost identical to that in the rat and other eutherian mammals. The region surrounding the 3' end of Rev-erbα is also highly conserved and is flanked upstream and downstream by homopolymeric runs of 3 or more C (C-rich or C_n_) and G (G-rich or G_n_) residues, respectively, as is the case in other mammals (Figure 1B and additional file 2).

Closer inspection of the sequence for *M. domestica *TRα, however, reveals that the first coding exon of TRα1 mRNA is entirely missing in the genome assembly due to a 5 kb gap [36]. Also, apparent sequencing errors at positions 54 and 124 of exon 6 disrupt the reading frame for TRα1 by introducing, respectively, a one-base insertion and a stop codon. The complete coding sequence for *M. domestica *TRα1 was obtained by sequencing cDNA using primers complementary to conserved sequences in the 5' UTR and at intervals throughout the coding sequence. The TRα1 coding sequence for *M. domestica *from this analysis was identical in all other respects to the current genome assembly (additional file 1 Panel A). As shown in Figure 1 the amino acid sequence of opossum TRα1 is closely similar to that of rat, with 93% identity at the amino acid level. Rev-erbα is also well conserved with 84% identity. The 3' ends of TRα1 and Rev-erbα mRNAs from opossum were mapped via RNase protection assays and 3' RACE, respectively (results not shown), and were found to coincide precisely with the sites previously reported for rat [15,32] as indicated in additional file 2. To confirm these observations, mRNA sequences for TRα1 and Rev-erbα were determined for a second marsupial, *Potorous tridactylus *(the long-nosed potoroo). The opossum and potoroo amino acid sequences, representing the two largest marsupial orders, Didelphia and Diprotodontia, about 80 My diverged [37], are very similar: 99% and 96% identity for TRα1 and Rev-erbα, respectively (additional file 1).

**Figure 2:**
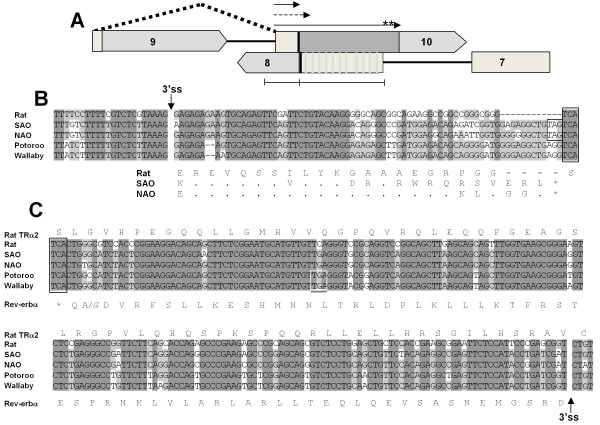
**Organization of sequence homologous to Exon 10 of TRα2**. **A**. Overlap between terminal exons of TRα2 and Rev-erbα mRNAs as in Figure 1. Light vertical lines in Rev-erbα coding sequence represent >30 synonymous substitutions within exon 8 of Rev-erbα. Thin horizontal arrows represent C-terminal polypeptides potentially or actually translated from exon 10 of TRα2. Dotted arrow indicates frame-shifted translation product of potoroo and wallaby. Asterisk on longest arrow indicates position of phosphorylation sites on rat TRα2. Double brackets under figure mark sequences highlighted in panels B and C. **B**. Alignment of 3'ss and 5' end of exon 10. Nucleotide sequence from rat and four marsupials is shown. The amino acid sequence corresponding to exon 10 of TRα2 is shown for the rat and the two opossums. Dashes indicate gaps and dots residues identical to sequence above. Boxes indicate stop codons in-frame with the 3' splice sites for either TRα2 (sense strand) or Rev-erbα (antisense strand). **C**. Alignment of overlapping coding sequences for TRα2 and Rev-erbα. The amino acid sequence for the rat TRα2 sequence (sense strand) is shown above the nucleotide sequence and the conserved amino acid sequence of Rev-erbα is shown at the bottom. Stop codons are indicated for Rev-erbα (black box, repeated from panel B) and that hypothesized for TRα2 of wallaby and potoroo (white box) assuming use of the homologous 3'ss splice site. The single amino acid substitution in the potoroo Rev-erbα sequence is indicated in italics (A/*G*). The stop codon for Rev-erbα is shown boxed in both alignments.

In contrast to the strong conservation of TRα1 and Rev-erbα, sequences corresponding to the alternatively spliced exon 10 of TRα2 mRNA display a puzzling mix of conserved and non-conserved features. Sequences corresponding to the core 3'ss elements for this exon are nearly identical in rat and *M. domestica*, including the intronic polypyrimidine tract and the non-consensus AAG immediately preceding the 3'ss (Figure 2B and additional file 2). Sequence at the 5' end of exon 10, corresponding to 11 codons in rat TRα2, is also well conserved but at that point the similarity between marsupial and eutherian sequence ends abruptly until reaching the coding sequence for Rev-erbα. The divergent region includes a 12 nucleotide insertion in opossum and an in-frame stop codon immediately preceding the stop codon for Rev-erbα on the strand antisense to TRα. These adjacent stop codons, present in both species of opossum but absent in wallaby and potoroo, as described below, precisely eliminate the bidirectional coding overlap between TRα2 and Rev-erbα mRNAs seen in rat and other eutherian mammals [17,38]. Finally, the Rev-erbα amino acid sequence encoded by its terminal exon is identical in opossum and rat. However, there are 26 synonymous substitutions in Rev-erbα sequence, almost all of which alter the coding potential of the TRα strand in opossum.

To further explore the significance of this sequence comparison, we examined 1.5 kb of genomic sequence that span the 3' end of TRα1 and extend across exon 8 and part of exon 7 of Rev-erbα in three other marsupial species: *Didelphis virginiana *(the Virginia opossum)*, P. tridactylus*, and *Macropus eugenii *(the tammar wallaby). Alignment of all four marsupial sequences with those of rat shows a high level of conservation of marsupial features similar to that shown in Figure 1B and additional file 2. As expected, since both are of the order Didelphimorphia, the sequence of the opossum *D. virginiana *is very similar to that of *M. domestica *(89% identity across mostly non-coding sequence). Both include a 12-nucleotide insertion and in-frame stop codon in the region adjacent to the stop codon for Rev-erbα (Figure 2B). Sequences for wallaby and potoroo (both of the order Diprotodontia) are similar to those from the opossums in the region homologous to exon 10 of TRα2. Furthermore, the amino acid sequences encoded by the 3' exon of Rev-erbα in all four marsupials are identical to that of rat except for a single residue change in the potoroo, although each sequence contains many substitutions which are silent with respect to Rev-erbα (Figure 2C).

Despite their high degree of similarity overall, the potoroo and wallaby sequences differ from those of the opossums in one notable respect: there is a two-basepair deletion immediately downstream of sequences homologous to the 3'ss of exon 10 in TRα2 mRNA. This deletion disrupts the reading frame corresponding to the C-terminus of TRα2 and creates a novel open reading frame that extends past the Rev-erbα stop codon, overlapping the Rev-erbα coding region by 16 codons (Figure 2B and 2C).

### Expression of TRα and Rev-erbα mRNAs in marsupials

Given the conservation of so many features within the TRα/Rev-erbα locus in marsupials, it seemed likely that marsupials might express an alternatively spliced mRNA yielding a truncated version of TRα2. To characterize expression of mRNAs from Rev-erbα and TRα genes we examined RNA from multiple tissues from *M. domestica *at six different ages ranging from newborn animals (0 days) to weanlings (9 weeks) and young adults (18 weeks).

**Figure 3:**
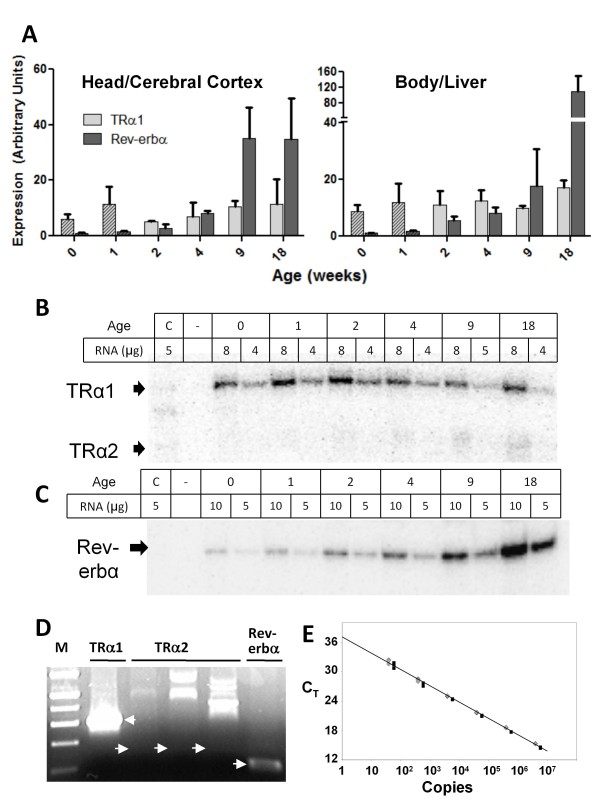
**Expression of TRα1, Rev-erbα and TRα2 mRNAs in the opossum *Monodelphis domestica***. **A**. Expression of TRα1 and Rev-erbα in staged animals is shown for heads and bodies (0 days and one week of age, hatched bars) or cerebral cortex and liver (2-18 weeks, evenly shaded bars) based on realtime RT-PCR analysis. Results are averages from at least three animals with each assay performed in triplicate. Brackets indicate standard deviations. **B and C**. RNase protection assays of TRα (**B**) and Rev-erbα (**C**) expression in heads (0 days and one week) or cerebral cortex (2-18 weeks). Probes extend across exon 9A (TRα1) or the 5'ss of exon 6 (Rev-erbα) as indicated in Figure 1A, yielding protected bands of 156 or 137 nts for TRα1 or Rev-erbα, respectively. Control lanes include HEK 293 cell RNA which contains a low level of endogenous human RNA that cross-reacts with the opossum TRα probe (compare with a similar control with the opossum probe in Fig. 4D). Arrows indicate positions of the expected products. **D**. Electrophoretic analysis of realtime RT-PCR reactions illustrating absence of TRα2 expression in *M. domestica*. Analysis of reactions run on same cDNA sample (day 0 heads) with arrows indicating positions of amplification products for TRα1 and Rev-erbα mRNAs and positions of product expected from correctly spliced TRα2 mRNA. Three replica reactions for TRα2 show a variety of artifactual products but no detectable product of the expected size. **E**. Amplification of a synthetic TRα2 template based on homologous sequences from opossum. Threshold cycle values (C_T_) are shown for serial dilution of test DNA in the presence of a constant amount of cDNA from day 0 heads (which has no detectable TRα2). Efficiencies of 99.4% and 99.6% were calculated from two independent preparations of the gel-purified synthetic template.

Expression of both TRα1 and Rev-erbα mRNAs was readily detected at all stages by RT-PCR. Since newborn and one-week animals are only 10 mm and 16 mm in length, respectively, these animals were simply divided into two parts, heads and bodies, for extraction of RNA. Beginning with two-week animals, six different tissues were collected: cerebral cortex, cerebellum, liver, kidney, skeletal muscle and heart. For each tissue at each stage, both TRα1 and Rev-erbα expression were readily detected by realtime RT-PCR and expression of both mRNAs was confirmed by sequencing PCR amplification products. Results of these assays are summarized in Figure 3A for heads and bodies of newborn (0 or 1 week), and cerebral cortex and livers of older animals (2-18 weeks). Similar results are shown for four other tissues in additional file 3. Increasing expression of Rev-erbα correlates strongly with increasing age (with a linear correlation coefficient, r > 0.95 in skeletal muscle, kidney, cerebellum and liver, and r> 0.74 in brain and heart). The highest levels of Rev-erbα expression were observed in liver and skeletal muscle; the lowest levels were in heart. In all tissues except liver Rev-erbα showed a large, 4- to 6-fold increase in expression between 4 and 9 weeks (p < 0.001). In fact, TRα1 expression also showed a strong, if less pronounced, correlation with increasing age in all tissues except heart (r ≥ 0.95 for TRα1 in kidney and cerebellum and r ≥ 0.74 in brain, muscle and liver) (additional file 3).

In contrast to TRα1 and Rev-erbα mRNA expression, both realtime and conventional RT-PCR assays for TRα2 were consistently negative. To confirm this result, multiple pairs of primers were tested, including forward primers in exons 7, 8 and 9 and reverse primers annealing to different sites within the region homologous to TRα2 exon 10 and Rev-erbα exon 8. While the threshold values obtained for TRα1 were highly reproducible, typically varying by <0.2 cycles for a given set of replicas, replica samples run with primers for TRα2 were highly variable, either displaying large threshold values or altogether failing to cross the threshold. Melt-curves (not shown) and gel electrophoresis of these products revealed a heterogeneous array of non-specific products, as illustrated in the replica lanes shown in Figure 3D. Additional file 4 summarizes threshold values from realtime RT-PCR for TRα2 across all tissues and ages (including the testes from 2 and 4 month animals) and shows parallel values for TRα1 for comparison. Two different downstream primers for TRα2 were used for most of these assays. In none of the assays was a product obtained consistent with splicing of TRα2 mRNA in opossum.

To test the efficiency and sensitivity of the TRα2 primers, realtime RT-PCR was carried out with a short synthetic DNA template matching the predicted amplicon sequence of opossum TRα2 mRNA. Serial dilutions of this template were assayed in the presence of a fixed amount of a negative cDNA preparation of *M. domestica *total RNA from cerebral cortex. The plot in Figure 3E indicates that these TRα2-specific primers have a high efficiency, >99% [39], and a sensitivity at C_T _= 33 of less than 50 copies of TRα2 cDNA.

Expression of TRα and Rev-erbα mRNAs was also measured by RNase protection assays on total RNA from the head (day 0 and week 1 animals) or cerebral cortex (2-18 week animals) as shown in Figure 3B. From the specific activity of the probe, the level of TRα1 mRNA was determined to vary between 0.3 and 0.7 amol/μg RNA (1 amol = 10^-18 ^moles) with no clear stage-specific trend. On the other hand, Rev-erbα increased steadily from less than 0.1 amol/μg RNA in day 0 animals to about 1.0 amol/ug RNA in 18 week animals (Figure 3C), a trend paralleling the increase observed by realtime RT-PCR in cerebral cortex and several other tissues (Figure 3A and additional file 3). TRα1 expression was assayed with a probe that extended across the 3' and 5' splice sites for exon 9A. The length of the protected fragment for TRα1 reveals efficient splicing of the former but not the latter site. In older opossums (9 and 18 weeks) a faint band corresponding in size to that expected for use of the 5'ss of TRα2 was observed. The presence of this band raises the possibility that an alternatively spliced product with a similar 5'ss to TRα2 is expressed in older animals. However, further efforts to identify such a product were unsuccessful.

### Requirements for splicing of TRα2

**Figure 4:**
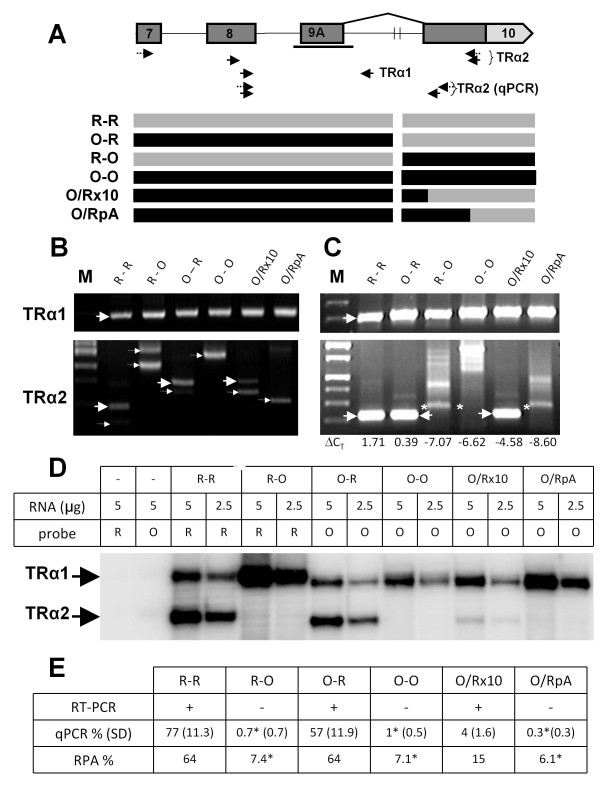
**Splicing of chimeric TRα minigenes**. **A**. Schematic showing intron-exon structure of TRα minigene. Light shading indicates the TRα2 3'UTR. Vertical brackets indicate a 3.2 kb deletion in intron 9. Heavy horizontal line represents the exon 9A RNase protection probe (panel D). Arrows represent primers used for PCR (panels B and C) as described in Methods. Primer positions for TRα2 mRNA are indicated separately for rat (solid arrows) and opossum (dashed arrows); those for TRα1 are located at nearly identical positions. The composition of chimeric minigenes is indicated by horizontal bars aligned with structure at top: dark and light shading represents opossum and rat sequences, respectively. **B, C**. Electrophoretic analysis of conventional (**B**) or realtime (**C**) RT-PCR of RNA from transfected HEK 293 cells using primers indicated in Panel A. Large arrows indicate PCR products from correctly spliced TRα1 or TRα2 mRNAs. Dotted arrows indicate spliced products obtained from cryptic splicing within exon 10. Larger products seen with TRα2 primers from R-O, O-O and O/RpA represent readthrough of unspliced or partially spliced RNAs, low levels of contaminating DNA (Panel B) or non-specific products (Panel C). ΔC_T _values shown in Panel C represent differences observed between threshold cycles (C_T_) for TRα1 and TRα2 (ΔC_T _= C_T_^TRα1 ^- C_T_^TRα2^) averaged over three replica assays from each of three independent experiments. **D**. Analysis of minigene splicing by RNase protection assays using probes for exon 9A. **E**. Summary of results in panels B, C and D. Plus signs represent reactions where correct splicing of TRα2 was detected following sequencing of conventional PCR products. % splicing was calculated from ΔC_T _values (see Methods) with standard deviations given in parentheses. Asterisks represent possible false positives from non-specific RT-PCR products or high background in RNase protection assays.

Although we found no evidence of TRα2 expression in *M. domestica*, the possibility remained that its expression is tightly restricted to some tissue, developmental stage or particular set of physiological conditions. To examine the intrinsic competency for splicing of conserved sequences corresponding to the 3'ss of exon 10 of TRα2, an opossum minigene was constructed based on the structure of a rat minigene that efficiently expressed TRα2 mRNA in transfection assays [32,40]. The construct consisted of exons 7-10 of TRα, extending downstream to exon 7 of Rev-erbα, with all the intron sequences except for a large deletion within intron 9 that eliminated the competing TRα1 polyadenylation site and much of the preceding 3' UTR (Figure 4A). This minigene was efficiently expressed in transient transfection assays in HEK 293 cells. RNA processing was assayed for both spliced (TRα2) and partially spliced ("unspliced" TRα1-like) RNA by conventional PCR (Figure 4B) and realtime RT-PCR (Figure 4C), as well as RNase protection assays (Figure 4D). Bands corresponding to spliced TRα2 were evident for the rat minigene (R-R) in all three assays. Real-time PCR and RNase protection assays yielded quantitative measures of TRα2 splicing relative to unspliced TRα1 of 64-77%, consistent with earlier results for this minigene construct. Conventional PCR employing larger amplicons reveals a minor amount of a second spliced product corresponding to the minor spliced isoform, TRα3 (small arrow, Figure 4B). In contrast, no spliced product could be detected from the opossum minigene (O-O). The larger band evident in Figure 4B (lane O-O) corresponds to readthrough sequence, while the heterogeneous bands in Figure 4C represent non-specific amplification products observed after 44 cycles of amplification. Close examination of the RNase protection assays (Figure 4D) revealed no bands corresponding to splicing of exon 9A. Figure 4E summarizes the results of each of the assays. A low level of residual background activity was routinely detected with RNase protection assays and real-time PCR yielding heterogenous amplification products, as indicated by asterisks in Figure 4E.

Given the efficiency of TRα2 splicing in the rat construct (R-R), the complete lack of expression of TRα2 splicing with the opossum minigene (O-O) was surprising. Both the 5' and 3' splice sites corresponding to TRα2 are highly conserved in the rat and the opossum (Figure 2B and additional file 2), as are intronic sequences adjacent to 5' and 3' splice sites. Despite the high conservation of these sequences in marsupials and eutherians, the opossum TRα gene may lack cis-acting elements required for TRα2 expression [32,38,40]. To extend this analysis chimeric minigenes were constructed and assayed for their ability to express TRα2. One construct, O-R, consisting of opossum exons 7-9 upstream of the rat TRα2 3' splice site and exon10, expressed TRα2 transcripts at levels similar to the rat minigene (Figure 4B, C, and 4D). Sequencing of the RT-PCR product obtained from transfected cells confirmed that this product was accurately spliced from the homologous 5' splice site in exon 9 of opossum to exon 10 of rat. In contrast, the reciprocal construct (R-O) with rat sequences upstream (exons 7-9) and opossum sequences downstream (exon 10) was apparently unable to express spliced TRα2 mRNA. In all of the constructs tested, the upstream exons 7, 8 and 9 are efficiently and accurately spliced, but only in O-R, not O-O or R-O, is the downstream splice site corresponding to exon 10 efficiently used. These results demonstrate that sequences homologous to the TRα2 5' splice site in exon 9 of opossum are fully competent for splicing, while those homologous to the TRα2 3'ss in exon 10 are not.

To more precisely define cis-acting elements required for TRα2 splicing, two additional minigenes were tested. The first of these, designated O/Rx10, was constructed from the O-R chimera by replacing the rat sequence upstream of the TRα2 3' splice site but downstream of intron 9 deletion with the opossum sequence present in the O-O minigene. The other chimeric minigene, O/RpA, was constructed by incorporating opossum sequences into the length of exon 8, thus eliminating the rat 3'ss for TRα2 while retaining the rat TRα2 polyadenylation site (Figure 4A). When these chimeric minigenes were transfected into HEK 293 cells the efficiency of TRα2 splicing was greatly reduced or eliminated. Careful analysis revealed that the O/Rx10 minigene expressed a small amount of correctly spliced chimeric TRα2 mRNA, as indicated by both RPA and RT-PCR assays, but with greatly reduced efficiency, strongly suggesting that opossum sequences in the intron upstream of exon 10 are incompatible with TRα2 splicing.

Post-run analysis of both R-O and O/RpA real-time products yielded a weak band in the region expected for correctly spliced product as indicated by the asterisks in Figure 4C. However, the difference between TRα1 and TRα2 C_T _values (ΔC_T _in Figure 4C) was comparable or less than that observed for the O-O minigene. Correct splicing of these transcripts could not be confirmed by other assays. Cryptic splicing within the opossum sequence in O/RpA, 60 nt from the chimeric junction of opossum and rat sequence, was detected by sequencing a band obtained by conventional PCR. Conventional PCR also demonstrated that R-R, O-R and O/Rx10 all express a small amount of spliced TRα3, a minor variant of TRα2 that uses an alternative 3'ss 117 bp downstream of the TRα2 3'ss in exon 10 [41]. For R-R and O-R this represents a minor product, slightly smaller than that for TRα2. For O/Rx10, however, this band is proportionately more intense, indicating a loss of specificity as well as a greatly reduced efficiency of TRα2 splicing for the O-Rx10 minigene.

The analysis described above strongly suggests that opossum sequences upstream of the 3'ss are incompatible with TRα2 splicing. The discrepancy between TRα2 splicing of O-R and O/Rx10 indicates that the TRα gene of *M. domestica *lacks upstream cis-acting elements required for efficient splicing of TRα2. Furthermore, inclusion of the rat TRα2 polyadenylation site, which is not conserved in marsupial species (additional file 2), is insufficient to restore accurate and efficient splicing in chimeric constructs O/Rx10 and O/RpA.

### Expression of endogenous and transfected mRNAs in PtK1 cells

**Figure 5:**
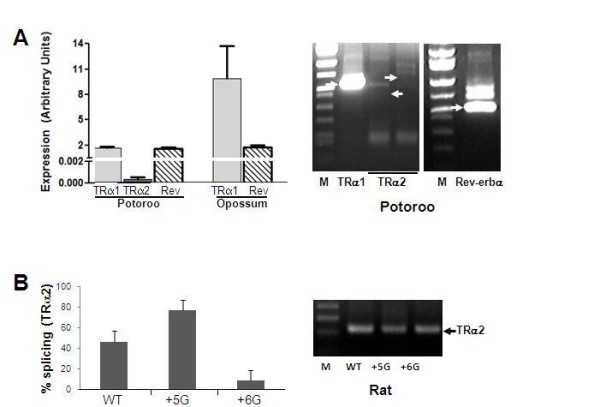
**Expression of TRα and Rev-erbα in potoroo PtK1 cells**. **A**. Endogenous expression of TRα1, TRα2 and Rev-erbα mRNAs was assayed by realtime RT-PCR (left; average of 3 replicas) and conventional PCR (right). Expression of TRα1 and Rev-erbα mRNAs in the opossum, *M. domestica *(day 0 heads), was measured in a parallel by realtime RT-PCR for comparison. Arrows on photograph at right indicate positions of expected amplification products for each mRNA. Two different primer pairs were targeted against TRα2, which was not observed. The predicted products for TRα1 and Rev-erbα mRNAs were confirmed by sequencing. The Rev-erbα RT-PCR matches sequence obtained from PtK1 cell DNA (additional file 2). No TRα2 mRNA was observed either by realtime RT-PCR or two sets of primers located in exon 7 or 8 and exon 10. **B**. TRα2 splicing of rat minigenes in PtK1 cells. Rat minigenes with full-length versions of exons 7-10 were expressed inPtK1 cells and assayed by realtime RT-PCR. Two previously characterized single-base point mutations in the 5'ss of exon 9A [32] were analyzed in parallel with wild-type and found to significantly overexpress (+5G) or underexpress (+6G) spliced TRα2 in comparison with wildtype (p < 0.05).

Analysis of the splicing of chimeric minigenes demonstrates that the opossum sequences homologous to the 3'ss of TRα2 splicing are not used efficiently. This most likely reflects the lack of cis-acting elements in opossum that are necessary for TRα2 expression [38,40]. It is possible, however, that the failure of opossum tissues to express TRα2 reflects the presence or absence of a factor that specifically affects TRα2 processing. To test this possibility we examined the processing of the rat TRα minigene in potoroo PtK1 cells. These cells, derived from kidney epithelium, endogenously expressed both TRα1 and Rev-erbα, but not TRα2 (Figure 5A). PtK1 cells were then transfected with three previously characterized full-length rat minigenes [32]: a wildtype minigene extending uninterrupted from exon 7 through exon 10 of TRα2 (and further downstream to exon 2 of Rev-erbα); a mutant with a single base replacement at the 5'ss of exon 9A (+5C/G), which overexpresses TRα2 mRNA; and a second mutant at that 5'ss with substitution at the adjacent site (+6T/G), which results in a decrease in TRα2 mRNA. When transfected into PtK1 cells, each of these constructs expressed TRα1 and TRα2 mRNAs at levels expected from previous expression studies in rodent and human cells (Figure 5B) [32]. These results demonstrate that marsupial cells expressing only endogenous TRα1 are fully capable of splicing rat TRα2.

## Discussion

Results of these studies show that TRα1 and Rev-erbα mRNAs are widely expressed in opossum in a manner similar to that in rodents, where both receptors are readily detectable in most tissues throughout development [20,42,43], with expression peaking in post-natal, pre-weanling mice and rat. Since marsupials are born at a relatively early stage of development and mature more slowly than rats and mice, the ages used here correspond roughly to late pre-natal through post-weanling stages in rats. The striking increase in the expression of Rev-erbα in most tissues with increasing age is also consistent with that reported for rat [44]. However, the failure to detect expression of TRα2 in a broad range of tissues at ages ranging from day 0 newborns to 4 month-old young adults contrasts strongly with the co-expression of all three of these mRNAs from this locus in rats, mice and other mammals.

The expression of any one of these mRNAs, TRα1, TRα2 or Rev-erbα, is almost always accompanied by expression of the other two, although the relative ratios vary widely [20,21,33]. Our analysis focused on the expression of these receptors in the central nervous system, as TRα2 mRNA is expressed at much higher levels than TRα1 in both cerebral cortex and cerebellum [42,43]. For example, in newborn rat brain TRα2 mRNA is more than 30-fold more abundant than TRα1 [42]. Furthermore, TRα2 expression in rats is several fold greater than TRα1 expression in brain, liver and kidney at all ages examined (day 15 embryos to adult) [42], a striking contrast to the total absence of TRα2 mRNA observed here in opossum and PtK1 cells.

### Conservation of sequences in TRα and Rev-erbα

The conservation of sequence spanning the 3' ends of TRα1 and Rev-erbα has several features of interest with respect to the evolution of TRα2 and the antisense overlap with Rev-erbα. The amino acid sequence encoded by the 3' exon of Rev-erbα is nearly identical in marsupial and eutherian mammals (100% identity in rat and opossum), suggesting that the coding sequence of Rev-erbα accommodated the evolution of its antisense overlap with TRα2 within its functional constraints as the coding sequence for TRα2 threaded through 67 (antisense) codons of Rev-erbα and into the final intron of the Rev-erbα gene. Interestingly, the one specific function ascribed to the unique C-terminal sequence of TRα2, that associated with phosphorylation of certain serine residues [45,46], maps to positions in TRα2 that are encoded antisense to the final intron of Rev-erbα and are therefore not constrained by requirements for Rev-erbα coding as shown in Figure 2A.

The region in which the 3' ends of TRα1 and Rev-erbα mRNAs converge is framed by exceptionally conserved sequences within the 3'UTR of each mRNA (additional file 2 ), suggesting the presence of numerous functional elements important for the expression and regulation of both nuclear receptor proteins. The apparent conservation of core splice site elements important for TRα2 expression is puzzling, given the apparent absence of TRα2 mRNA in marsupials, and raises the question of whether these elements correspond to TRα2 or have other functions. In particular, several features are consistent with conservation of the alternatively spliced TRα2-specific exon in marsupials: (1) conservation of the non-canonical AAG trinucleotide identical to that found in all known eutherian genomes at the 3' end of exon 9 for TRα2; (2) the conservation of adjacent sequences corresponding to the 5' end of exon 10; and (3) the conservation of the polypyrimidine tract that is an essential feature of the core sequence of the 3'ss. In fact, the marsupial polypyrimidine tract appears a better match to the mammalian consensus sequence than that found in rat: a G at position -6 is replaced with T in all four marsupials and a somewhat higher proportion of T/C residues is present in the marsupial polypyrimidine tracts [47,48].

However, other features of this region are consistent with the absence of TRα2 in opossum: (1) the open reading frame corresponding to the extended C-terminus of TRα2 is not conserved in four marsupials except for the first 11 codons of TRα2 exon 10 in the opossums; (2) the bidirectional coding overlap with Rev-erbα present in all eutherian species is eliminated in both opossums; (3) the TRα2 ORF is disrupted in wallaby and potoroo by a frame-shift deletion adjacent to the presumptive splice site; (4) a conserved polyadenylation signal corresponding to exon 10 of TRα2 is absent, and there is overall poor conservation of sequence within the final intron of *Rev-erbα *(additional file 2); and (5) there is poor conservation of intronic and exonic splicing enhancer sequences required for TRα2 splicing [38,40]. These include the SEα2 enhancer downstream of the coding sequence for TRα1 [40], an intronic splicing enhancer near the 3' splice site of exon 10 and a exonic enhancer located within the bidirectional coding sequence of exon 10 [38]. Taken together, these differences suggest that the conservation of the elements associated with TRα2 mRNA processing may reflect other functional requirements at this locus unrelated to TRα2 expression.

### Splicing of TRα2 mRNA in chimeric minigenes

Analysis of splicing of opossum and rat minigenes corroborated our finding that alternatively spliced TRα2 mRNA is missing in marsupials. Most telling was the O/Rx10 construct, which includes the entire length of rat exon 10, identical to that in O-R, except for replacement of 180 bp of rat sequence upstream of the TRα2 3'ss with 220 bp of opossum sequence. The longer opossum sequence closely corresponds to a previously tested construct (ErbAΔXP) which was spliced as efficiently as the shorter sequence [38]. The overall level of splicing of O/Rx10 was <10% that of O-R and the predominant spliced product corresponded to TRα3, a minor splice variant isoform, not TRα2, as shown by sequencing of RT-PCR products (Figure 4B). These experiments demonstrate that opossum sequences homologous to the TRα2-specific 3'ss are almost completely inactive in splicing transfected minigenes. In addition to the role of these intronic sequences in blocking TRα2 splicing, the opossum sequences also lacked a conserved polyadenylation signal hexanucleotide (CATAAA) that is conserved in all known TRα2 mRNAs. However, the inclusion of this element (together with much additional rat sequence) in the O/Rx10 and O/RpA minigenes was not sufficient to rescue efficient splicing.

### Evolution of TRα2 mRNA in mammals

A number of studies suggest that the evolution of new alternative exons provides an important pathway for the creation of novel proteins and regulatory pathways in eukaryotic cells [49-52], reviewed in [53]. Given the prominence of TRα2 as an abundant isoform in eutherian mammals [42], its proposed role as a dominant negative modulator of thyroid hormone activity [19] and the possible regulatory implications of its overlap with Rev-erbα [33], the evolution and function of TRα2 in mammals are of particular interest.

**Figure 6:**
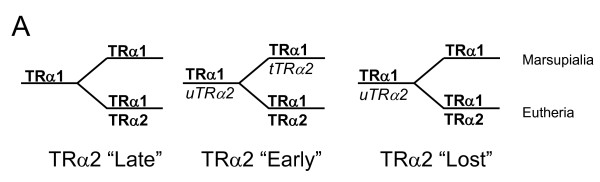
**Models for the evolution of antisense exon 10 in TRα and expression of the TRα2 isoform**. Three models are proposed for the evolution of the TRα2 isoform and its antisense overlap with Rev-erbα. In the first model (TRα2 "late"), alternatively spliced TRα2 mRNA evolved only after the divergence of marsupial and eutherian mammals so that TRα2 is unique to eutherian mammals. In the second model (TRα2 "early"), a functional but primitive version of TRα2 (*uTRα2*) evolved early in the course of mammalian evolution, but in marsupials the modern, truncated form of TRα2 (*tTRα2*) is expressed only at very low levels or its expression is temporally or spatially restricted. In the third model (TRα2 "lost"), a functional version of TRα2 (uTRα2) evolved very early in the common ancestor of marsupial and eutherian mammals, but this alternative isoform was lost at some later time in marsupial lineage.

Three distinct evolutionary scenarios or models may account for similarities and differences of this locus in marsupial species as summarized in Figure 6. The first scenario, in which TRα2 expression is restricted to the eutherian lineage, suggests that some features of the marsupial TRα/Rev-erbα locus are intermediate between those of eutherian mammals and non-mammalian vertebrates. For example, the poly(A) sites of TRα1 and Rev-erbα are nearly identically spaced in rat and opossum and much closer than in the amphibian *Xenopus tropicalis*, where they are more than 10 kb apart [38]. On the other hand, marsupials, like non-mammalian vertebrates, appear to lack an authentic TRα2 isoform. Also, the amino acid sequence of the marsupial TRα1 (additional file 1 Panel A) shows striking similarities to that of chicken. The opossum TRα1 amino acid sequence differs from chicken in only 22 positions as compared to 25 positions in rat (additional file 1 Panel A), despite a slightly greater similarity between the opossum and rat nucleotide coding sequences. In 20 of the 25 positions where opossum and rat TRα1 differ, the opossum sequence is identical to that of chicken. Most of these sites are also conserved in frog, a more distantly related vertebrate (result not shown). These observations suggest that the marsupial TRα locus has retained several characteristics reminiscent of non-mammalian vertebrates.

The second scenario, in which a truncated form of TRα2 is expressed in some limited (though as yet undetected) fashion, also suggests that the marsupial locus may represent a stage intermediate between the more complex eutherian locus, with its extended antisense overlap with Rev-erbα and abundant expression of TRα2, and the non-mammalian vertebrates, which entirely lack TRα2. Support for this model is found in the fact that many features characteristic of the marsupial TRα/Rev-erbα locus are associated with the creation of novel alternatively spliced exons, according to several recent studies [49-53]. These include inefficient splicing coupled with restricted or low levels of expression, as posited in the second model, and the absence of an extended open reading frame.

The third scenario, that TRα2 evolved early but was lost in the marsupial lineage, suggests that features characteristic of TRα2 that are shared by marsupial and eutherian mammals are vestiges of this loss. These vestigial features may have acquired other secondary functions within this locus. Although this scenario may appear overly complex and hence unlikely, careful analysis of nuclear receptor evolution throughout multiple metazoan phyla reveals that loss within a particular lineage is not at all uncommon [54]. Among approximately 25 nuclear receptor proteins that appear to have been present in the last common ancestor of vertebrate and bilaterian invertebrate species, a number of nuclear receptors, including both thyroid hormone receptor genes, are missing entirely from major groups of animals such as nematodes or arthropods while specific orthologs for both thyroid hormone receptors and Rev-erb are found in more distantly related animals, such as mollusks, tunicates and flatworms [55-57].

Although none of the three models can be definitively ruled out, on balance, the first scenario might be judged most likely. The failure to detect TRα2 expression in tissues and stages where it is abundantly expressed in eutherians, the poor efficiency of TRα2 minigene splicing, the absence of a discernible TRα2 polyadenylation signal element and differences between the presumptive coding sequence for TRα2 in two marsupial orders weigh against the second model. The differences and similarities among the marsupial sequences within the 3' UTR of Rev-erbα (Figure 2 and additional file 2), including strict conservation of 3' processing sites for TRα1 and Rev-erbα, seem most consistent with the first model, in which sequence conservation reflects regulatory requirements for these convergent mRNAs rather than vestiges of a once functional, alternatively spliced mRNA, as suggested in the third model.

If the first scenario is correct, apparent similarities between marsupials and eutherians in the region corresponding to the TRα2 3' ss and exon 10 must reflect other conserved functions. The first model thus suggests core elements of the TRα2 splice site were coopted (or "exapted") [58,59] during the evolution of TRα2. What, then, are the possible functions of these sequences? It is likely that they reflect the overlay of elements involved in regulating expression of TRα and Rev-erbα, as well as other possible roles. Accumulating evidence suggests that sequences at the 3' ends of genes play multiple roles in regulating gene expression. These include target sites for microRNA binding [60], sites for binding of proteins regulating transcription termination, 3' end processing, turnover and translation [61,62], and promoters for expression of non-coding transcripts that are often found within the 3' UTRs of transcripts [1,3,7].

### Physiological correlates of nuclear receptor function

The physiological role of TRα2 has remained elusive since its discovery more than two decades ago, despite numerous genetic and molecular investigations of its function [63-65]. Although many studies have focused on its role as a weak dominant negative competitor of TRα1 or TRβ isoforms [19,66-68], other studies have pointed to its phosphorylation-dependent RNA-binding activity and cytoplasmic localization [46,69,70]. These properties, along with the developmental regulation of its expression, suggest a role for TRα2 in developmental regulation of T3 activity [71,72]. The present study strongly indicates that its function is specific to the eutherian lineage, and thus may ultimately be resolved by comparative analysis of relevant aspects of marsupial and eutherian physiology.

The multiple roles ascribed to Rev-erbα and TRα1 in metabolic, circadian and developmental signaling [22,27,73-75] require a diversity of regulatory inputs that determine the expression and physiological activity of these receptors [20,21]. The evolution of the antisense overlap between TRα2 and Rev-erbα mRNAs in eutherian mammals is likely to be associated with additional mechanisms that affect the expression or activity of both receptors.

## Conclusions

Analysis of the TRα/Rev-erbα locus in four marsupial species strongly suggests that the alternatively spliced TRα2 isoform, which is widely and abundantly expressed in all known eutherian mammals, is not present in marsupials. Three observations support this conclusion: first, we can find no evidence for TRα2 expression in any of multiple tissues or developmental stages surveyed; second, the coding sequence specific to TRα2 is severely truncated in opossums and completely altered in the wallaby and potoroo; and third, opossum sequences homologous to the 3'ss specific for TRα2 are utilized very inefficiently for splicing of chimeric minigenes. The antisense overlap between TRα2 and Rev-erbα mRNAs, which is tightly conserved in eutherian mammals, appears to have evolved within the constraints of the conserved C-terminal coding sequence of Rev-erbα. These results suggest that comparative analysis of these genes in marsupials and eutherian mammals will provide further insight into the evolution, function and expression of TRα2 and the regulatory implications of its antisense overlap with Rev-erbα.

## Methods

### Tissues and cells

*Monodelphis domestica *tissues were harvested from staged animals obtained from stocks maintained at the Southwest Foundation for Biomedical Research. The opossums were maintained under standard conditions for this species [76]. The Southwest Foundation for Biomedical Research is accredited by the Association for the Assessment and Accreditation of Animal Care, International. All procedures were approved by the Institutional Animal Care and Use Committee and conformed to the Public Health Service Policy on Humane Care and Use of Laboratory Animals. DNA was collected from animals at birth (0 days), 1, 2 and 4 weeks, 2 months (9 weeks) and 4 months (18 weeks). DNA from *Didelphis virginiana *was isolated from a purchased sample of brain tissue (Pel-Freez; Rogers, AR). *Potorous tridactylus *PtK1cells (CRL-6493) were obtained from ATCC (Manassas, VA) and passaged in Dulbeccos modified Eagles medium with 10% fetal bovine serum.

### Nucleic acid sequencing and cloning

DNA was sequenced from the overlap regions spanning the TRα and Rev-erbα genes of *Didelphis virginiana *and *Potorous tridactylus *following amplification of total DNA using primers complementary to conserved sites within exon 9 of TRα1 and exons 7 and 8 of Rev-erbα. mRNA sequencing was similarly carried out with cDNA obtained by randomly primed reverse transcription of total RNA (Superscript II; Invitrogen) following homogenization of tissue in TRIzol according to manufacturer's instructions (Invitrogen). Amplified genomic products were also cloned into pGEM-T vector (Promega; Madison, WI) and subcloned into pErbA construct as previously described [40]. PCR amplifications were carried out with Taq polymerase (Promega). All primers were from Integrated DNA Technologies (Coral, IA)

Minigene plasmid constructs are based on the rat construct, pErbAΔXE (R-R in Figure 4). Both the rat and possum constructs include all but the first 20 bp of exon 7, and all of exons 8, 9A and 10 [32,38]. In this plasmid intron 9 sequences are deleted from an XbaI site 490 bp downstream of the 5'end of exon 9 in rat and a site 186 bp upstream of the exon 10 3'ss. Sequences of both rat and opossum minigenes extend to a unique BstEII site in Rev-erbα exon 7 that is conserved in *M. domestica*. The opossum minigene, pErbA(O-O) ("O-O" in Figure 4) includes 620 bp of intron 9 upstream of the deletion and 226 nts downstream following insertion of an XbaI site to facilitate swapping of rat and opossum sequences in construction of the chimeric plasmids pErbA(O-R) and pErbA(R-O) (Figure 4A). Chimeric plasmids pErbA(O/RpA) and pErbA(O/Rx10) were prepared by two-step recombinant PCR as previously described [38]. Wild-type plasmid pCMV-ErbAm and the corresponding 5'ss point mutants, +5C/G and +6T/G, have been previously described [32].

The following DNA and mRNA sequences (with NCBI accession) were analyzed using Vector NTI software (Invitrogen): *M. domestica *NW_001581875.1, XM_001370259, XM_001362861; *Ratus novegicus *NW_047339, NM_001017960, NM_031134, NM_001113422; *Gallus gallus *NM_205313. The *Macropus eugenii *genomic sequence was assembled from the following files in the trace archives: ti:1070151041, ti:1437108730, ti:1378054157, ti:1582562684, ti:1623537866, ti:1484317168, ti:1467742023, ti:1070151372.

The following sequences have been deposited in the GenBank: *M. domestica *TRα1 mRNA (HM149330), *P. tridactylus *TRα1 (HM149329), *P. tridactylus *NR1D1 (HM149328), *P. tridactylus *TRα1/Rev-erbα genomic sequence (HM149331), *D. virginiana *TRα1/Rev-erbα genomic sequence (HM149332). The *M. eugenii *TRα1/Rev-erbα genomic sequence has been deposited in the NCBI Bankit database (BK007078).

### Transfection assays and RNA analysis

Plasmids were transfected into HEK 293 cells using calcium phosphate as described [32] and into PtK1 cells with Lipofectamine 2000 (Invitrogen). Total RNA was prepared from transfected cells with TRIzol 48 h post-transfection. RNA was assayed by RNase protection assays as previously described [32,33]. Riboprobes for rat TRα1/TRα2 exon 9 and Rev-erbα exon 5 have been previously described [32]. Similar riboprobes were prepared for opossum Rev-erbα exon 5, TRα1 exons 9A, and TRα1 poly(A) site by in vitro transcription of PCR products cloned into pGEM-T vector (Promega) using primers described in (additional file 5). The riboprobe for opossum, unlike that for rat, extended upstream into intron 8 as well as downstream of the site homologous to exon 9A in TRα2 (Figure 4A). 3'RACE was carried out on RNA from liver and cerebral cortex from 4 month animals using gene specific primers for opossum Rev-erbα. Direct sequencing of PCR products obtained with two gene-specific primers confirmed the presence of a major poly(A) site coincident with that found in rat, human and mouse in the EST database.

Quantitative realtime RT-PCR was carried out as follows. Total RNA was randomly primed and reverse transcribed with Superscript II. For expression assays with possum tissues, 4 ug of total RNA was used per reaction and the equivalent of 0.1 μg of RNA product was used in a 25 μl reaction containing Bio-Rad SybrGreen Supermix and 150 nM primers (additional file 5 ). RNA from transfected cells was similarly treated except less RNA was used. PCR values from opossum tissues were normalized to expression of opossum β-actin with primer to exons 5 and 6. Realtime PCR assays were carried out on a MyiQ Realtime Thermocycler using IQ SyberGreen Supermix and analyzed according to the manufacturer's instructions (Bio-Rad Laboratories; Hercules, CA).

The following primers were used for amplification of chimeric gene products as shown in Figure 4C, with expected amplicon lengths (in base pairs) given in parentheses: for TRα1 in O-O, O-R, O/Rx10 and O/RpA: 1F/2R (290); for TRα1 in R-R and R-O: 15F/16R (291); (2) for TRα2 in O-O and O/RpA 1F/6R (271); for TRα2 in O-R and O/Rx10 1F/17R (222); for TRα2 in R-O 15F/6R (251); and for TRα2 in R-R 15F/17R (202). For conventional PCR shown in Figure 4B (40 cycles) the same primers for TRα1 but for TRα2 longer amplicons were used: O-O and O/RpA 20F/21R (722); O-R and O/Rx10 20F/23R (728); R-R 22F/23R (471); and R-O 22F/21R (464).

The efficiency of TRα2 primers was determined from the slope of the semilogarithmic plot of template concentration vs. C_T _using the equation, efficiency = [10^(-1/slope)] -1 [39]. As a further test of primers used to probe for TRα2 expression, the "forward" TRα2 primer was paired with a "reverse" primer specific for TRα1, while the "reverse" TRα2 primers were paired with a "forward" primer specific to the 3' UTR of Rev-erbα. Each of these primer combinations efficiently amplified the expected mRNAs, supporting the conclusion that the failure to detect TRα2 mRNA does not reflect shortcomings of primer design (data not shown). Relative expression of TRα1 and TRα2 and percent splicing of TRα2 were calculated from realtime RT-PCR assuming 100% amplification efficiency. The significance of changes in expression was evaluated by Student's two-sample unpooled t test.

## Authors' contributions

SHM and BCR initiated this study in consultation with JLV; BCR and MSB performed the experiments and SHM, MSB and BCR wrote the manuscript. All authors approved the final manuscript.

## Supplementary Material

Additional file 1 **Alignment of TRα1 mRNAs for *R. norvegicus*, *M. domestica*, *P. tridactylus *and *G. gallus***. **A**. TRα1 mRNA. The amino acid sequence for Gallus gallus (chick) TRα1 is included below that for R. novegicus (rat), *M. domestica *(SAO) and *P. tridactylus *(potoroo). **B**. Rev-erbα mRNA. Start and stop codons are boxed in red as is the 5' splice site for TRα2 in exon 9 of TRα1 in panel A. The boundary between exons 8 and 9 is indicated in panel B with a vertical line. Click here for file

Additional file 2 **Alignment of genomic sequence showing overlap region for rat, *M. domestica *(SAO), *D. virginiana *(NAO), *P. tridactylus *(potoroo) and *M. eugenii *(wallaby)**. The alignment is annotated to show various features including polyadenylation sites and splice sites. The boundaries for subregions indicated in Figure 1B are also shown (vertical lines):. Red boxes indicate in-frame stop codons and polyadenylation signal sequences. Dotted red boxes in marsupial sequences show those stop codons inframe with the TRα2-specific splice site (as in Figure 2). Red arrows mark splice sites and polyadenylation sites. Click here for file

Additional file 3 **Expression of TRα1 and Rev-erbα in cerebellum, kidney, skeletal muscle and heart expression isolated from 2 to 18 week old opossums**. Tissues from 2, 4 9 and 18 week old opossums (*M. domestica*) were assayed for TRα1 and Rev-erbα expression as in Figure 3A. Results are averages of three animals, with each assay performed in triplicate. Brackets indicate standard deviations. Click here for file

Additional file 4 **Table showing realtime RT-PCR data illustrating absence of detectable TRα2 mRNA in opossum tissues**. Uncorrected threshold values (C_T _values) are shown for TRα2 mRNA in upper left of the box for each age and tissue. For comparison, values for TRα1 in parallel runs are shown the lower right of each box. Each value is the average of 3 replica determinations from a single run, with samples failing to reach the threshold arbitrarily set to 42 in determining the average. Boxes outlined in bold show runs where measurements for TRα1 and TRα2 were carried out simultaneously. All samples were assayed for TRα2 with two different primer sets. Values shown without asterisks were measured with the primer pair evaluated in Figure 3E. Asterisks indicate measurements with an alternate TRα2 primer pair also used in Figure 3D. Replica values for TRα1 show little variation (SD< 0.2 for all but 4 measurements). C_T _values for TRα2 typically showed high values ( > 38) and/or substantial variation (SD > 1) characteristic of non-specific products over a 44 cycle run. Click here for file

Additional file 5 **Table of primers used for PCR, DNA sequencing and plasmid constructions**. Click here for file
