# Supplementary material for: Structure and expression of two nuclear receptor genes in marsupials: insights into the evolution of the antisense overlap between the α-thyroid hormone receptor and Rev-erbα
Source: BMC Mol Biol. 2010 Dec 10;11:97. doi: 10.1186/1471-2199-11-97 (PMC3047299; doi:10.1186/1471-2199-11-97)
Supplement: Additional file 1 — Alignment of TRα1 mRNAs for R. norvegicus, M. domestica, P. tridactylus and G. gallus. A. TRα1 mRNA. The amino acid sequence for Gallus gallus (chick) TRα1 is included below that for R. novegicus (rat), M. domestica (SAO) and P. tridactylus (potoroo). B. Rev-erbα mRNA. Start and stop codons are boxed in red as is the 5' splice site for TRα2 in exon 9 of TRα1 in panel A. The boundary between exons 8 and 9 is indicated in panel B with a vertical line. [file 1471-2199-11-97-S1.PDF]

Additional figure 1A

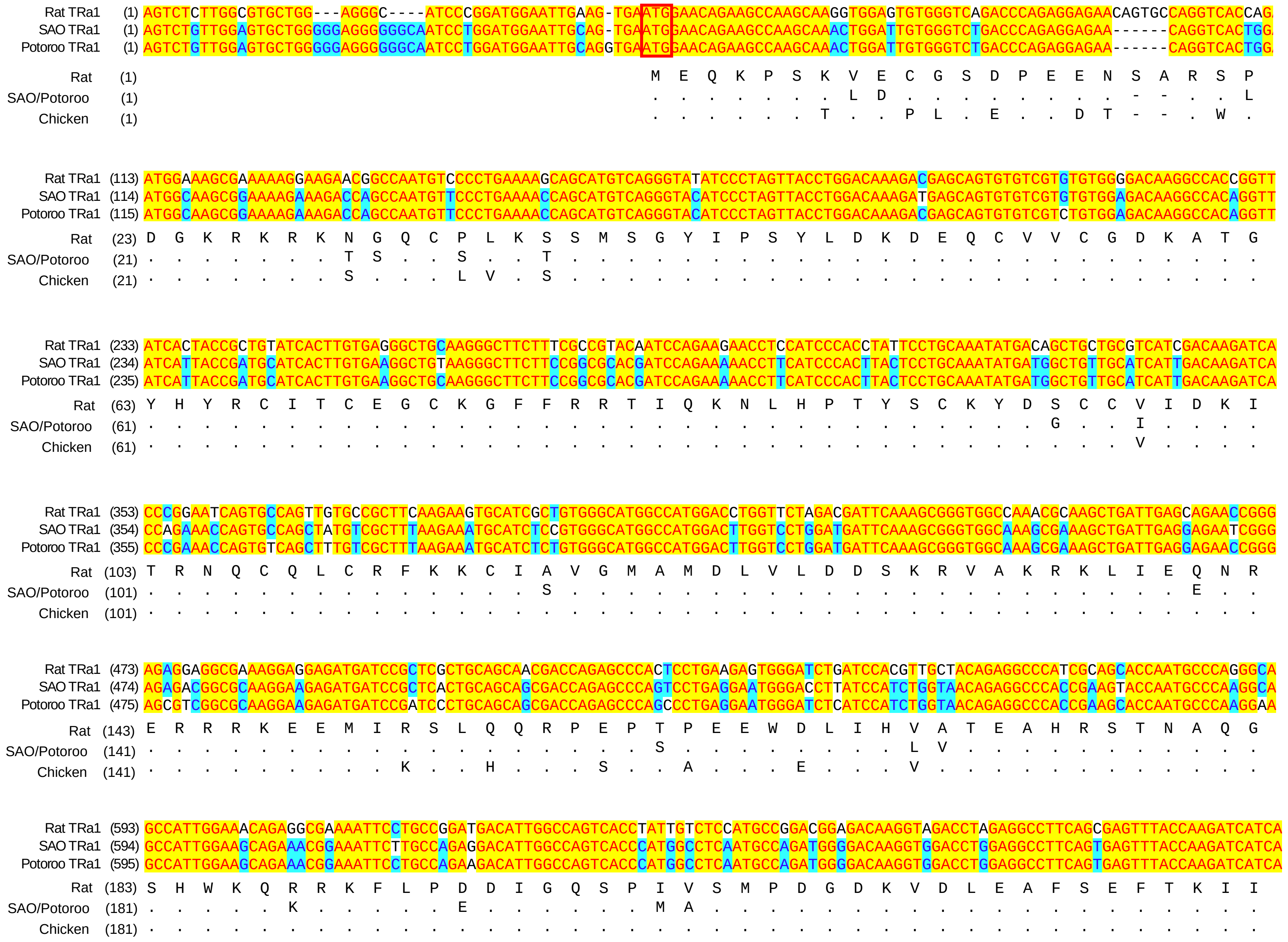

Additional figure 1A (continued)

```
Rat TRa1      (713)  CCCCGGCCATCACCCGCGTGGTGGACTTTGCCAAAAAACTGCCCATGTTCTCCGAGCTGCCTTGTGAAGACCAGATCATCCTCCTGAAGGGCTGCTGCATGGAGATCATGTCCCTGCGGG
SAO TRa1      (714)  CCCCGGCCATCACCCGTGTGGTGGACTTTGCCAAAAAACTGCCCATGTTCTCCGAGCTGCCTTGTGAAGACCAGATCATCTTGCTGAAGGGCTGCTGCATGGAGATCATGTCTCTTCGGG
Potoroo TRa1  (715)  CCCCGGCCATCACCCGTGTGGTGGACTTTGCCAAAAAACTGCCCATGTTCTCCGAGCTGCCTTGTGAAGACCAGATCATCTTACTGAAAGGCTGCTGCATGGAGATCATGTCTCTGCGAG

Rat           (223)  T P A I T R V V D F A K K L P M F S E L P C E D Q I I L L K G C C M E I M S L R
SAO/Potoroo   (221)  . . . . . . . . . . . . . . . . . . . . . . . . . . . . . . . . . . . . . . . .
Chicken       (221)  . . . . . . . . . . . . . . . . . . . . . . . . . . . . . . . . . . . . . . . .

Rat TRa1      (833)  CAGCTGTCCGCTATGACCCTGAGAGCGACACCCTGACCCTGAGTGGGGAGATGGCGGTTAAGCGGGAGCAGCTCAAGAATGGTGGCTTGGGTGTAGTCTCCGACGCCATCTTTGAACTGG
SAO TRa1      (834)  CCGCAGTAAGATATGATCCTGAGAGTGAGACTTTGACGCTGAGTGGGGAGATGGCAGTCAAACGGGAGCAGCTGAAGAATGGTGGCCTGGGGGTGGTCTCTGATGCCATCTTCGATCTGG
Potoroo TRa1  (835)  CTGCCGTACGCTATGACCCCGAGAGTGAGACTTTAACGCTGAGTGGGGAGATGGCAGTCAAACGGGAGCAGCTGAAGAATGGCGGCCTGGGGGTGGTCTCCGATGCCATCTTCGACCTGG
Rat           (263)  A A V R Y D P E S D T L T L S G E M A V K R E Q L K N G G L G V V S D A I F E L
SAO/Potoroo   (261)  . . . . . . . . . E . . . . . . . . . . . . . . . . . . . . . . . . . . . . D .
Chicken       (261)  . . . . . . . . . . . . . . . . . . . . . . . . . . . . . . . . . . . . . . . .

Rat TRa1      (953)  GCAAGTCACTCTCTGCCTTTAACCTGGATGATACGGAAGTGGCTCTGCTGCAGGCTGTGCTGTTAATGTCAACAGACCGCTCTGGCCTGCTGTGTGTGGACAAGATCGAGAAGAGTCAGG
SAO TRa1      (954)  GCAAGTCACTCTCTGCCTTCAACCTGGATGACACAGAAGTGGCTCTGCTCCAGGCTGTGCTGCTCATGTCTTCAGACCGCTCGGGCCTGCTGTGTGTGGACAAGATCGAGAAGTGCCAGG
Potoroo TRa1  (955)  GCAAGTCACTCTCTGCCTTCAACCTGGATGACACGGAAGTCGCTCTGCTTCAGGCTGTGCTGCTCATGTCTTCAGACCGCTCGGGCCTGCTGTGTGTGGACAAGATCGAGAAGTGCCAGG
Rat           (303)  G K S L S A F N L D D T E V A L L Q A V L L M S T D R S G L L C V D K I E K S Q
SAO/Potoroo   (301)  . . . . . . . . . . . . . . . . . . . . . . . . S . . . . . . . . . . . . . C .
Chicken       (301)  . . . . . . . . . . . . . . . . . . . . . . . . . . . T . . I . . . . . . . . .

Rat TRa1     (1073)  AGGCCTACCTGCTGGCGTTTGAGCACTACGTCAACCACCGCAAACACAACATTCCGCACTTCTGGCCCAAGCTGCTGATGAAGGTGACTGACCTCCGCATGATCGGGGCCTGCCACGCCA
SAO TRa1     (1074)  AGGCTTACCTGCTGGCTTTCGAACACTACATCAACCATCGCAAACACAACATTCCCCACTTCTGGCCCAAGCTGTTGATGAAGGTGACTGACCTCCGGATGATAGGGGCCTGCCATGCCA
Potoroo TRa1 (1075)  AGGCTTACCTGCTGGCTTTTGAACACTACATCAACTACCGCAAACACAACATTCCCCACTTCTGGCCCAAGCTGCTGATGAAGGTGACTGACCTCCGGATGATCGGGGCCTGCCATGCCA
Rat           (343)  E A Y L L A F E H Y V N H R K H N I P H F W P K L L M K V T D L R M I G A C H A
SAO/Potoroo   (341)  . . . . . . . . . . I . H/Y . . . . . . . . . . . . . . . . . . . . . . . . . .
Chicken       (341)  . T . . . . . . . . . . Y . . . . . . . . . . . . . . . . . . . . . . . . . . .

Rat TRa1     (1193)  GCCGCTTCCTCCACATGAAAGTCGAGTGCCCCACCGAACTCTTCCCCCACTCTTCCTGGAGGTCTTTGAGGATCAGGAAGTCTAAAGCCTCAGGCGGCCAGAGGGTGTGCGGAGCTGG-
SAO TRa1     (1194)  GCCGCTTCTTACACATGAAAGTCGAGTGCCCCACTGAACTCTTTCCCCCGCTCTTCCTTGAGGTCTTCGAGGATCAGGAAGTCTAAGCCCCCAG--TGCTGGGGTATGGGGAGAACTGGG
Potoroo TRa1 (1195)  GCCGGTTCCTACACATGAAAGTCGAGTGCCCCACCGAACTCTTTCCCCCTCTCTTCCTTGAGGTCTTCGAGGATCAGGAAGTCTAAACCCCTAG--TGCTGGGGGATGAGGAGAGGTGGG
Rat           (383)  S R F L H M K V E C P T E L F P P L F L E V F E D Q E V *
SAO/Potoroo   (381)  . . . . . . . . . . . . . . . . . . . . . . . . . . . . *
Chicken       (381)  . . . . . . . . . . . . . . . . . . . . . . . . . . . . *
```

Additional figure 1B

```
Rat      (1)   GAATGACCGCTTTCAGCTGGTGAAGACATGACGACC---CTAGACTCCAACAACAACACAGGTGGTGTTATCACCTACATTGGCTCCAGCGGATCCTCCCCCAGCCGGACCAGCCCAGAG
SAO      (1)   GAATGAGAGCTGTGAGTTTGTGAAGACATGACGACCACTCTGGACTCCAACAATAATACAGGTGGAGTCATCACCTACATTGGTTCCAATGGCTCATCTCCCAGCCGAACCAGCCCTGTT
Potoroo  (1)   GAATGAGAGCTGTCAGTTGGTGAAGACATGACGACCACTCTGGACTCCAACAATAACACAGGTGGAGTCATCACCTACATCGGTTCCAATGGTTCATCTCCTAGCCGAACCAGCCCTGTT
Rat      (1)   M T T - L D S N N N T G G V I T Y I G S S G S S P S R T S P E
SAO      (1)   . . . T . . . . . . . . . . . . . . . . N . . . . . . . . . V
Potoroo  (1)   . . . . . . . . . . . . . . . . . . . . . . . . . . . . . . .
```

```
Rat      (118) TCCCTCTATAGTGACAGCTCCAATGGCAGCTTCCAGTCCCTGACTCAAGGTTGTCCCACATACTTCCCACCATCACCTACTGGCTCCCTCACCCAGGACCCTGCCCGCTCATTTGGCACT
SAO      (121) TCTCTCTGCAGTGAGAGCTCCAATGGCAGCTTCCAGTCCCTGACCCACGCCTTTCCTACCTATTTTCCACCCTCCCCCACTGGCTCCCTCACCCAGGACCCAGGCCGACCCTTTGGGGGT
Potoroo  (121) TCTCTCTGCAGTGAGAGCTCCAATGGAAGCTTTCAGTCCCTGACCCATGCCTTCCCTACCTACTTTCCACCCTCCCCCACTGGCTCCCTCACCCAGGACCCAGGCCGGTCCTTTGGGAGT
Rat      (31)  S L Y S D S S N G S F Q S L T Q G C P T Y F P P S P T G S L T Q D P A R S F G T
SAO      (32)  . . C . E . . . . . . . . . . H A F . . . . . . . . . . . . . . . . G . P . . G
Potoroo  (32)  . . . . . . . . . . . . . . . . . . . . . . . . . . . . . . . . . . . . S . . S
```

```
Rat      (238) GTGCCACCCAGCCTCAGTGATGATAGCTCCCCT---------------TCTTCTGCTTCATCATCATCATCATCTTCCTCCTCCTCCTTCTATAATGGGAGCCCCCCGGAAGTCTACAA
SAO      (241) GGCCCCCCTGGCTTGAGGGAAGATGGCTCCCCTTCATCGTCATCTTCATCTTCTTCCTCCTCCTCTTCGTCTTCATCTTCTGCCTCCTTCTATAATGGAGGCTCCCCTGGAGGACTTCAA
Potoroo  (241) GGCCCTCCTGGCTTGAGAGAAGAAGGCTCCCCTTCA---TCATCATCATCTTCATCTTCCTCATCTTCCTCTTCTTCCTCTGCCTCCTTCTATAATGGAGGCTCCCCTGGAGGACTGCAG
Rat      (71)  V P P S L S D D S S P - - - - - S S A S S S S S S S S S S F Y N G S P P G S L Q
SAO      (72)  G . . G . R E . G . . S S S S S . . S . . . . . . . . A . . . . . G S . G G . .
Potoroo  (72)  . . . . . . . E . . . - . . . . . . . . . . . . . . . . . . . . . . . . . . . .
```

```
Rat      (343) GTGGCCATGGAAGACAGCAGCCGAGTGTCCCCCAGCAAGGGCACCAGCAACATTACCAAGCTTAACGGCATGGTGCTACTGTGTAAAGTGTGTGGGGACGTGGCCTCAGGCTTCCACTAT
SAO      (361) GTAGCTATGGAGGACAGTAGCCGAGTGTCCCCTAGCAAGAGTACCAGCAGCATCACCAAGCTCAATGGCATGGTGCTCCTTTGTAAGGTGTGTGGGGATGTTGCCTCTGGCTTCCACTAT
Potoroo  (358) GTGGCCATGGAGGACAGCAGCCGAGTATCCCCCAGCAAGAGTACCAGCAGCATCACCAAGCTCAATGGCATGGTGCTCCTTTGTAAAGTGTGTGGGGATGTTGCCTCCGGCTTCCATTAT
Rat      (106) V A M E D S S R V S P S K G T S N I T K L N G M V L L C K V C G D V A S G F H Y
SAO      (112) . . . . . . . . . . . . . S . . S . . . . . . . . . . . . . . . . . . . . . . .
Potoroo  (111) . . . . . . . . . . . . . . . . . . . . . . . . . . . . . . . . . . . . . . . .
```

```
Rat      (463) GGCGTGCATGCCTGCGAGGGCTGCAAGGGCTTTTTCCGCCGGAGCATCCAGCAGAACATCCAGTACAAGCGGTGTTTGAAAAACGAGAACTGCTCCATTGTCCGTATCAATCGCAACCGC
SAO      (481) GGAGTCCATGCCTGTGAAGGTTGCAAGGGCTTTTTCCGACGAAGCATCCAGCAGAACATCCAGTACAAAAAGTGCCTAAAGAATGAGAGTTGCTCCATCATCCGTATCAACCGTAACCGA
Potoroo  (478) GGAGTCCACGCTTGTGAAGGCTGCAAGGGCTTTTTCCGACGAAGCATTCAGCAGAACATCCAGTACAAAAAGTGCCTAAAAAATGAGAGTTGCTCTATCATCCGCATCAACCGTAACCGC
Rat      (146) G V H A C E G C K G F F R R S I Q Q N I Q Y K R C L K N E N C S I V R I N R N R
SAO      (152) . . . . . . . . . . . . . . . . . . . . . . . K . . . . . S . . . I . . . . . .
Potoroo  (151) . . . . . . . . . . . . . . . . . . . . . . . . . . . . . . . . . . . . . . . .
```

```
Rat      (583) TGCCAGCAGTGTCGCTTCAAGAAGTGTCTCTCCGTTGGCATGTCTAGAGACGCTGTGCGTTTTGGACGTATCCCCAAGAGAGAGAAGCAACGAATGCTTGCTGAGATGCAGAACGCCATG
SAO      (601) TGTCAGCAATGCCGATTCAAGAAATGCCTGTCTGTTGGCATGTCCCGAGATGCCGTGCGTTTTGGACGGATCCCAAAAAGAGAGAAGCAGAGGATGCTTGCTGAGATGCAAAGTGCCATG
Potoroo  (598) TGCCAGCAGTGCCGATTCAAGAAATGCTTGTCTGTTGGCATGTCCCGAGATGCTGTACGTTTTGGACGGATCCCAAAGAGAGAGAAGCAAAGGATGCTTGCCGAGATGCAAAGTGCCATG
Rat      (86)  C Q Q C R F K K C L S V G M S R D A V R F G R I P K R E K Q R M L A E M Q N A M
SAO      (192) . . . . . . . . . . . . . . . . . . . . . . . . . . . . . . . . . . . . . S . .
Potoroo  (191) . . . . . . . . . . . . . . . . . . . . . . . . . . . . . . . . . . . . . . . .
```

Additional figure 1B (continued)

```
Rat      (703)   AACTTGGCCAACAACCAACTGAGCAGCCTGTGCCCTCTAGAGACCTCACCTGCCCCGCACCCCACCTCAGGCTCCGTGGGCCCTCACCACCTCCTGCACCAGCCCCGACACCTTTGGTG
SAO      (721)   AACCTGGCTAATAACCAACTGAGCACTCAGTGCCCACCTGAGGCCTCACCTGCCAGGATCCCTGCACCTGGCCCACTGGGCTCCTCACCACCCCCAGTTCCAGCTCCCTCACCTCTTGTG
Potoroo  (718)   AACCTGGCTAATAATCAGCTGAGCACTCAGTGCCCACCTGAAGCCTCACCTGCCAGAATCCCTCCCTCTGGCCCTTTGGGCTCCTCCCCACCTCCGGTTCCAGCTCCCTCACCCCTTGTG
Rat      (226)    N  L  A  N  N  Q  L  S  S  L  C  P  L  E  T  S  P  A  P  H  P  T  S  G  S  V  G  P  S  P  P  P  A  P  A  P  T  P  L  V
SAO      (232)    .  .  .  .  .  .  .  .  T  Q  .  .  P  .  A  .  .  .  R  I  .  A  P  .  P  L  .  S  .  .  .  .  V  .  .  .  S  .  .  .
Potoroo  (231)    .  .  .  .  .  .  .  .  .  .  .  .  .  .  .  .  .  .  .  .  .  P  S  .  .  .  .  .  .  .  .  .  .  .  .  .  .  .  .  .

Rat      (823)   GGCTTCTCTCAGTTCCCACAACAGCTGACACCACCCAGATCCCCGAGTCCTGAGCCCACCGTGGAGGATGTGATATCCCAGGTGGCCAGGGCCCATCGAGAAATCTTCACCTATGCCCAT
SAO      (841)   GGCTTTTCCCAGTTCCCCCAGCAGTTGACACCACCCCGATCTCCAAGTCCTGAGGCCAAAGTGGAGGATGTCATCTCCCAGGTGGCCCGAGCCCACAGAGAGATCTTCACCTATGCTCAT
Potoroo  (838)   GGTTTCTCCCAGTTTCCCCAGCAGCTGACACCACCTCGATCTCCAAGTCCTGAGGCCAAAGTGGAGGATGTGATCTCCCAGGTGGCTCGAGCCCACAGAGAGATCTTCACCTATGCCCAT
Rat      (266)    G  F  S  Q  F  P  Q  Q  L  T  P  P  R  S  P  S  P  E  P  T  V  E  D  V  I  S  Q  V  A  R  A  H  R  E  I  F  T  Y  A  H
SAO      (272)    .  .  .  .  .  .  .  .  .  .  .  .  .  .  .  .  .  .  A  K  .  .  .  .  .  .  .  .  .  .  .  .  .  .  .  .  .  .  .  .
Potoroo  (271)    .  .  .  .  .  .  .  .  .  .  .  .  .  .  .  .  .  .  .  .  .  .  .  .  .  .  .  .  .  .  .  .  .  .  .  .  .  .  .  .

Rat      (943)   GACAAATTAGGCACCTCACCTGGCAACTTCAATGCCAATCATGCATCAGGTAGCCCTCCGGCTACCACTCCACAGTGCTGGGAGAGTCAGGGATGCCCGTCT---ACCCCCAACGACAAC
SAO      (961)   GACAAGTTGGGAACTGCACCCAATGGCCTGAATGCCAACCAGACACCCAGTGGCCCCCCAACATCTACAGTACCCCGCTGGGAAAATCATTGCTGTCCTTCAGCACCTCCTGATGACAAC
Potoroo  (958)   GACAAGCTGGGCACTACACCCAGTGGCCTGAATGCCAACCAGACGCCCAACGGCCCCCCAACATCTACAACACCCCGCTGGGAAAACCATTGCTGTCCTCCAGCACCTCCTGATGACAAC
Rat      (306)    D  K  L  G  T  S  P  G  N  F  N  A  N  H  A  S  G  S  P  P  A  T  T  P  Q  C  W  E  S  Q  G  C  P  S  -  T  P  N  D  N
SAO      (312)    .  .  .  .  .  A  .  N  G  L  .  .  .  Q  T  P  S  G  .  .  T  S  .  V  P  R  .  .  N  H  C  .  .  .  A  P  .  D  .  .
Potoroo  (311)    .  .  .  .  .  T  .  S  .  .  .  .  .  .  .  .  N  .  .  .  .  .  .  T  .  .  .  .  .  .  .  .  .  P  .  .  .  .  .  .

Rat     (1060)   AACCTTTTGGCGGCTCAGCGTCATAATGAAGCACTGAATGGTCTACGCCAGGGCCCCTCCTCCTACCCTCCTACCTGGCCTTCTGGCCCTGCCCACCACAGCTGCCACCAGCCTAACAGC
SAO     (1081)   AACTCAGCTGCCACCCAGCGCCACAATGAAGCCCGAAATGGACTTCATCCAGCTCCAAACTCATACCCACCAACCTGGCCAACTGCTCCTTCCCATCATGGATGCCACCAGCATAATAGC
Potoroo (1078)   AACTCTGCTGCCACCCAGCGCCACAATGAAGCCCGAAATGGACTTCGTCCAGCTCCAAACTCATACCCACCAACCTGGCCAACTGCTACCCCCCACCATGGGTGCCACCAGCATAATAGC
Rat      (345)    N  L  L  A  A  Q  R  H  N  E  A  L  N  G  L  R  Q  G  P  S  S  Y  P  P  T  W  P  S  G  P  A  H  H  S  C  H  Q  P  N  S
SAO      (352)    .  S  A  .  T  .  .  .  .  .  .  R  .  .  .  H  P  A  .  N  .  .  .  .  .  .  .  T  A  .  S  .  .  G  .  .  .  H  .  .
Potoroo  (351)    .  .  .  .  .  .  .  .  .  .  .  .  .  .  .  R  .  .  .  .  .  .  .  .  .  .  .  .  .  T  P  .  .  .  .  .  .  .  .  .

Rat     (1180)   AATGGGCATCGCCTGTGCCCCACCCACGTATACTCGGCCCCAGAAGGCAAGGCACCTGCCAACGGTCTACGGCAAGGCAACACCAAGAATGTTCTGCTGGCATGTCCCATGAACATGTAT
SAO     (1201)   AATGGGCATCGCCTCTGCCCTACCCACATGTATCAAACCCCAGAGGCTGAAACCTCATCTGGTTGTCCCTGGCAGGACCGCTCTAAGAACATTCTTTTGGCATGCCCCATGAACATGTAC
Potoroo (1198)   AATGGGCACCGCCTCTGCCCTACTCACATGTATCAAACTCCAGAGGCTGAAACCTCAACTGGCTGCCCTTGGCAGGATCGATCTAAGAACATTCTATTGGCGTGCCCCATGAATATGTAC
Rat      (385)    N  G  H  R  L  C  P  T  H  V  Y  S  A  P  E  G  K  A  P  A  N  G  L  R  Q  G  N  T  K  N  V  L  L  A  C  P  M  N  M  Y
SAO      (392)    .  .  .  .  .  .  .  .  .  M  .  Q  T  .  .  A  E  T  S  S  G  C  P  W  .  D  R  S  .  .  I  .  .  .  .  .  .  .  .  .
Potoroo  (391)    .  .  .  .  .  .  .  .  .  .  .  .  .  .  .  .  .  .  .  T  .  .  .  .  .  .  .  .  .  .  .  .  .  .  .  .  .  .  .  .

Rat     (1300)   CCCCATGGACGTAGTGGCCGGACTGTGCAGGAGATCTGGGAAGACTTCTCTATGAGCTTCACACCCGCTGTGCGGGAGGTGGTAGAATTTGCCAAACACATCCCCGGCTTCCGTGACCTT
SAO     (1321)   CCCCATGGACGTAGTGGGCGAAGCGTTCAGGAGATCTGGGAGGACTTCTCAATGAGCTTCACACCTGCTGTGCGTGAAGTTGTAGAATTTGCTAAGCATATCCCAGGCTTCTGTGATCTC
Potoroo (1318)   CCCCATGGGCGCAGTGGGCGAAGTGTTCAGGAGATCTGGGAGGACTTCTCCATGAGCTTCACGCCTGCTGTTCGAGAAGTTGTAGAGTTTGCTAAGCATATCCCAGGCTTCTGTGACCTC
Rat      (425)    P  H  G  R  S  G  R  T  V  Q  E  I  W  E  D  F  S  M  S  F  T  P  A  V  R  E  V  V  E  F  A  K  H  I  P  G  F  R  D  L
SAO      (432)    .  .  .  .  .  .  .  S  .  .  .  .  .  .  .  .  .  .  .  .  .  .  .  .  .  .  .  .  .  .  .  .  .  .  .  .  .  C  .  .
Potoroo  (431)    .  .  .  .  .  .  .  .  .  .  .  .  .  .  .  .  .  .  .  .  .  .  .  .  .  .  .  .  .  .  .  .  .  .  .  .  .  .  .  .
```

Additional figure 1B (continued)

```
Rat      (1420) TCTCAGCACGACCAGGTGACCCTGCTTAAGGCTGGCACCTTTGAGGTGCTGATGGTGCGCTTTGCGTCATTGTTCAACGTGAAGGACCAGACAGTCATGTTCCTGAGCCGCACAACCTAC
SAO      (1441) TCTCAGCATGACCAGGTCACTCTGCTCAAGGCTGGTACCTTTGAGGTGCTGATGGTACGTTTCGCTTCACTGTTCAATGTGAAGGAGCAAACAGTTATGTTCCTGAGCCGCACAACATAC
Potoroo  (1438) TCTCAACACGACCAGGTCACTCTGCTCAAAGCTGGTACCTTTGAGGTGCTGATGGTACGTTTTGCTTCACTATTCAATGTGAAAGAACAAACGGTTATGTTCCTGAGCCGTACGACATAC

Rat      (465)   S  Q  H  D  Q  V  T  L  L  K  A  G  T  F  E  V  L  M  V  R  F  A  S  L  F  N  V  K  D  Q  T  V  M  F  L  S  R  T  T  Y
SAO      (472)   .  .  .  .  .  .  .  .  .  .  .  .  .  .  .  .  .  .  .  .  .  .  .  .  .  .  .  .  .  .  .  .  .  .  .  .  .  .  .  .
Potoroo  (471)   .  .  .  .  .  .  .  .  .  .  .  .  .  .  .  .  .  .  .  .  .  .  .  .  .  .  .  .  .  .  .  .  .  .  .  .  .  .  .  .
```

```
Rat      (1540) AGCCTGCAGGAGCTCGGTGCCATGGGCATGGGTGACCTGCTCAATGCCATGTTTGACTTCAGCGAGAAGCTCAACTCTCTGGCGCTTACTGAGGAGGAGCTGGGCCTTTTCACGGCAGTG
SAO      (1561) AGTCTGCAGGAACTGGGCGCCATGGGCATGGGTGACCTGCTCAATGCCATGTTCGACTTCAGTGAAAAGCTTAACTCCTTGGCCCTCACAGAAGAGGAGCTGGGACTGTTTACTGCAGTG
Potoroo  (1558) AGTCTGCAGGAACTGGGCGCTATGGGGATGGGGGACCTGCTCAATGCCATGTTCGACTTCAGTGAAAAGCTGAACTCCCTGGCCCTCACAGAAGAGGAGCTGGGGCTGTTTACTGCAGTA
Rat      (505)   S  L  Q  E  L  G  A  M  G  M  G  D  L  L  N  A  M  F  D  F  S  E  K  L  N  S  L  A  L  T  E  E  E  L  G  L  F  T  A  V
SAO      (512)   .  .  .  .  .  .  .  .  .  .  .  .  .  .  .  .  .  .  .  .  .  .  .  .  .  .  .  .  .  .  .  .  .  .  .  .  .  .  .  .
Potoroo  (511)   .  .  .  .  .  .  .  .  .  .  .  .  .  .  .  .  .  .  .  .  .  .  .  .  .  .  .  .  .  .  .  .  .  .  .  .  .  .  .  .
```

```
                     Exon 7          Exon 8
Rat      (1660) GTACTTGTCTCTGCAGACCGCTCGGGAATGGAGAATTCCGCTTCGGTGGAGCAGCTCCAGGAGACGCTGCTGCGGGCTCTTCGGGCTCTGGTGCTGAAGAACCGGCCCTCGGAGACTTCC
SAO      (1681) GTGCTTGTCTCTGCAGATCGATCAGGTATGGAGAACTCGGCCTCTGTAGAACAGCTGCAGGAGACGCTGCTCCGGGCTCTTCGGGCCCTGGTCCTGAAGAATCGGCCCTCAGAGACCTCC
Potoroo  (1678) GTGCTGGTCTCTGCAGACCGATCAGGTATGGAGAACTCAGCCTCTGTGGAACAGTTGCAGGAGACACTGCTCCGAGCACTTCGGGCACTGGTCCTAAAGAACAGGCCCTCAGAGACATCC
Rat      (545)   V  L  V  S  A  D  R  S  G  M  E  N  S  A  S  V  E  Q  L  Q  E  T  L  L  R  A  L  R  A  L  V  L  K  N  R  P  S  E  T  S
SAO      (552)   .  .  .  .  .  .  .  .  .  .  .  .  .  .  .  .  .  .  .  .  .  .  .  .  .  .  .  .  .  .  .  .  .  .  .  .  .  .  .  .
Potoroo  (551)   .  .  .  .  .  .  .  .  .  .  .  .  .  .  .  .  .  .  .  .  .  .  .  .  .  .  .  .  .  .  .  .  .  .  .  .  .  .  .  .
```

```
Rat      (1780) CGCTTCACCAAACTGCTGCTCAAGCTGCCGGACCTGCGGACCCTGAACAACATGCATTCCGAGAAGCTGCTGTCCTTCCGGGTGGACGCCCAGTGAC--CCGCCCGGCCG------GCCT
SAO      (1801) CGCTTCACCAAGCTGCTGCTTAAGCTGCCTGACCTGCGCACCCTCAACAACATGCATTCCGAGAAGTTGCTGTCCTTCCGAGTAGATGCCCAGTGACTACAGCCTCTCCACCGATCTCTG
Potoroo  (1798) CGCTTCACCAAGCTACTGCTTAAGCTGCCTGACCTCCGTACCCTCAACAACATGCATTCCGAGAAGCTGCTGTCCTTCCGAGTAGATGGCCAGTGACCTCAGCCTCCCCATCCCCTGCTG
Rat      (585)   R  F  T  K  L  L  L  K  L  P  D  L  R  T  L  N  N  M  H  S  E  K  L  L  S  F  R  V  D  A  Q  *
SAO      (592)   .  .  .  .  .  .  .  .  .  .  .  .  .  .  .  .  .  .  .  .  .  .  .  .  .  .  .  .  .  .  .  .
Potoroo  (591)   .  .  .  .  .  .  .  .  .  .  .  .  .  .  .  .  .  .  .  .  .  .  .  .  .  .  .  .  .  G  .  .
```
