# Supplementary material for: Structure and expression of two nuclear receptor genes in marsupials: insights into the evolution of the antisense overlap between the α-thyroid hormone receptor and Rev-erbα
Source: BMC Mol Biol. 2010 Dec 10;11:97. doi: 10.1186/1471-2199-11-97 (PMC3047299; doi:10.1186/1471-2199-11-97)
Supplement: Additional file 2 — Alignment of genomic sequence showing overlap region for rat, M. domestica (SAO), D. virginiana (NAO), P. tridactylus (potoroo) and M. eugenii (wallaby). The alignment is annotated to show various features including polyadenylation sites and splice sites. The boundaries for subregions indicated in Figure 1B are also shown (vertical lines):. Red boxes indicate in-frame stop codons and polyadenylation signal sequences. Dotted red boxes in marsupial sequences show those stop codons inframe with the TRα2-specific splice site (as in Figure 2). Red arrows mark splice sites and polyadenylation sites. [file 1471-2199-11-97-S2.PDF]

Additional figure 2

```
Rat     (1)   GGGAAGGAGGTAGACAGGTCCCTTTTCCCCAGAGATGGGTG--GGACCGGTAGGGGTGACTTGAGGGGCTCCTACTCCTGCCCCACA--G----TTGACAATCAGTATGTCTGTTAATGT
SAO     (1)   TGGCTGGTTGGGGGTAGCTCC-TTCCCCCTAGGGAGGGAAAATGGCT-GGTAGGAG-GAATGGGGGGAGGCCTA-TCCTGACCCCCACCCCATATTGACAATCAGTATGTCTGTTA-TGT
NAO     (1)   TGGCTGGTTGGGGGTAGCTCC-TTCCCCTTAGGGAGGGAAAATGGCC-GGTAGGAG-GAATGGGGGGAGGCCTA-TCCTGACCCCCACCCCATATTGACAATCAGTATGTCTGTTA-TGT
Potoroo (1)   TGGCTGGTTGGGGGTAGCTCC-TTCCCT-TAGGGAGGGATAATGGCC-GGTATAAG-GAATGGGGGGAGGCCTA-TCCTGACCCCCACCCCATTTTGACAATCAGTATGTCTGTTA-TGT
Wallaby (1)   TGGCTGGTTGGGGGTAGCTCC-TTCCCG-TAGGGAGGGATAATGGCCCGGTAGGAG-GAATGGGGGGAGGCCTA-TCCTGACCCCCACCCCATTTTGACAATCAGTATGTCTGTTA-TGT
```

```
                                                                                                TRα1 poly(A)         |C-rich
Rat     (113) GCGATTTTTCACCCCGTTGTGTTTTGGGTCAGGATTTTAAAGAAAGATATTTTTATGGTAATTGTTGCTCGTCTATTTTACTATATATTTATGTAATAAATATATGATGAAAATAAC-CC
SAO     (116) GCGATTTTTCACCCCGTTGTGTTTTGGGTCAGGATTTTAAAGAAAGATATTTTTATGGTAATTGTCGCTCGTCTATTTTACTATATATTTATGTAATAAATATATGATGAAAATAACTCC
NAO     (116) GCGATTTTTCACCCCGTTGTGTTTTGGGTCAGGATTTTAAAGAAAGATATTTTTATGGTAATTGTCGCTCGTCTATTTTACTATATATTTATGTAATAAATATATGATGAAAATAACTCC
Potoroo (115) GCGATTTTTCACCCCGTTGTGTTTTGGGTCAGGATTTTAAAGAAAGATATTTTTATGGTAATTGTCGCTCGTCTATTTTACTATATATTTATGTAATAAATATATGATGAAAATAACTCC
Wallaby (116) GCGATTTTTCACCCCGTTGTGTTTTGGGTCAGGATTTTAAAGAAAGATATTTTTATGGTAATTGTCGCTCGTCTATTTTACTATATATTTATGTAATAAATATATGATGAAAATAACTCC
```

```
Rat     (232) CCGGGCACCCCCCCTAGACTTGCGTGCTGTTTCCCCATATTCTCCCATCTGCTGGCAGAGTACCCACCCCACAAGCTGACCAGATGGAGAGGTGCCCCCCAACCCCCAGCCTTGGCAGTA
SAO     (236) CCTGAGAACCCTTCTGTGCCTGCATGGGCTAT----ATT-----CCTTCC---------------ACCCCA-AATCTG--CCAGTAGCCTAG-GCTCTCGATCCTGCAGGAGGTC---TC
NAO     (236) CCTGAGGACCCTTCTGTGCCTGCATGGGTTAT----ACTT--CTCCTTCT---------------ACCCCA-AATCTG--CTAGTAGCATAG-GCCCTCGACCCTGCAGGAGGTC---TC
Potoro  (235) CTTGTGGACCCTTCTAAGCCTGCATCTGCTGT----ATGTTGCCCCTTCT---------------ACCCCA-AATTTG--CAGGTAGAGTAG-GCCTTTGATCCAGAAGGCAGTA---TC
Wallaby (236) CTTGTGGACCCTTCTAAGCCTGCATCTGCTAT----GTGTTGCCCCTTCT---------------ACCCCA-AATTTG--CTGGTAGAGTAG-GCTTCTGATCCAGAAGGCAGTAGTATC
```

```
                   C-rich|                    Rev-erbα poly(A)                      |G-rich
Rat     (352) GTCCCCCCCCATTCCCCAAACGAGAGCACATCACAGAAGCCAGCTCAGC----TGTGAACTATTGGATTTGAGACAGGAACAGAACAAATCGAGGGGCCAGAGGAGGGTGGGAGAGCAAG
SAO     (325) CTCTTCCCCCCA-CCCCC-ACA----CACAGCACAGAAGCCAGATCAGATAGATGTCAGCTACTG-ATTTGAGATAG-AACAGGACACATC--------GGGTGATGGTG----------
NAO     (328) CTCTTCCCCCGA-CCCCC-ACA----CACAGCACAGAAGCCAGATCAGATAGATGTCAGCTACTG-ATTTGAGATAG-AACAGGACACATT--------GGGTGATGGTGG---------
Potoroo (329) CTCTTCCCCCCA-CCTCTAACA----CACACCACAGAAGCCATATCAGATAGATGTTAACTACTG-ATTTGAGATGGGAACAGGACACATC--------AGGTGAGGG------------
Wallaby (333) CTCTTCCCCCCA-CCCCAGACA----CACACCACAGAAGCCATATCAGATAGATGTCAACTACTG-ATTTGAGATGGGAACAGGACACATC--------AGGTGAGGG------------
```

```
                                                  G-rich|
Rat     (468) AGTGGTTTAAATACGGGAGG-AAGGGAGCATGGGGGTGGGGGGGAAGAGTTATTTACAAGAAGGCT---C----AGGGGG---------CCAGAGGCTCATCTTGGAATATTTTATAACA
SAO     (196) ---GGTTCAAC--AGGAAGG-GATAGAGCAGTTCCTTAAGGAGG--GAACTATTTACAAGAAGGCTGGGC-TGGAGGAGC---------CCAGAGGCTCTTCAGGGGATATTTTATAACA
NAO     (423) ---GGTTCAAC--AGGAAGG-GACAGAGCAGTTCCATAAGGAGG--GAGTTATTTACAAGAAGGCTGGGG-TGGAGGAGC---------CCAGAGGCTCATCAGGGGACATTTTATAACA
Potoroo (421) ---GGTTCAA---AGGAAGGAGAAAGAGCGGTTCCATGGGAAGG--GAGCTATTTACAAGAAGGCTGGGGGTGGAGGTGGGAGTAGGGGGCAGAGGCTCATCTTGGAATATTTTATAACA
Wallaby (423) ---GGTTCAA---AGGAAGGAGAAAGAGCAGTTCCATGGGAAGG--GAGCTATTTACAAGAAGGCTGGGG-TGGAGGTGGGAGTGGGGGCCACAGGCTCATCTTGGAATATTTTATAACA
```

```
                                             TRα2 3'splice site
Rat     (571) ATATAAATAAGATTCTGG--------------TTTGCTTTTCCTTTTCGTCTCGTAAAGGAGAGAGAAGTGCAGAGTTCGATTCTGTACAAGGGGCAGCGGCAGAAGGCCGGCCGGGC
SAO     (521) ATATAAATAAGATTCTGGGG-AAGGGACGG------TGTTTGTCTTTTTGTCTCTTAAAGAAGAGAGAAGTGCAGAGTTCAGTTCTGTACAAGGACAGGGCCCGATGGAGACAGAGATCG
NAO     (525) ATATAAATAAGATTCTGGGG-AAGGGATGG------TGTTTGTCTTTTTGTCTCTTAAAGGAGAGAGAAGTGCAGAGTTCAGTTCTGTACAAGGACAGGGCCCGATGGAGGCAGAAATTG
Potoroo (535) ATATAAATAAAATCCTGGGGAAAGGGGTGGTGTTGGTTTTATCTTTTTTGTCTCTTAAAGGAGAGA--AGTGCAGAGTTCAGTTCTGTACAAGGAGAGAGCTTGATGGAGACAGCAGGGG
Wallaby (538) ATATAAATAAGATCCTGGGGGAAGGGGTGGTGTTTGTTTTATCTTTTTTGTCTCTTAAAGGAGAGA--AATGCAGAGTTCAGTTCTGTACAAGGAGAGAGCTTGATGGAGACAGCAGGGG
```

```
Rat     (676) GGG------------TCACTGGGCGTCCACCCGGAAGGACAGCAGCTTCTCGGAATGCATGTTGTTCAGGGTCCGCAGGTCCGGCAGCTTGAGCAGCAGTTTGGTGAAGCGGGAAGTCTC
SAO     (634) GTGGAGAGGCTGTAGTCACTGGGCATCTACTCGGAAGGACAGCAACTTCTCGGAATGCATGTTGTTGAGGGTGCGCAGGTCAGGCAGCTTAAGCAGCAGCTTGGTGAAGCGGGAGGTCTC
NAO     (638) GTGGGGGGCTGTAGTCACTGTGCATCTACTCGGAAGGACAGCAGCTTCTCGGAATGCATGTTGTTGAGGGTGCGCAGGTCAGGCAGCTTAAGCAGCAGCTTGGTGAAGCGGGAGGTCTC
Potoro  (653) ATGGGGAGGCTGAGGTCACTGGCCATCTACTCGGAAGGACAGCAGCTTCTCGGAATGCATGTTGTTGAGGGTACGGAGGTCAGGCAGCTTAAGCAGTAGCTTGGTGAAGCGGGATGTCTC
Wallaby (656) ATGGGGAGGCTGAGGTCACTGGGCATCTACTCGGAAGGACAGCAGCTTCTCGGAATGCATGTTGTTGAGGGTACGGAGGTCAGGCAGCTTAAGCAGTAGCTTGGTGAAGCGGGAAGTCTC
```

Figure S2 (continued)

```
                                                                                                 Rev-erbα 3'splice site
Rat     (784)  CGAGGGCCGGTTCTTCAGCACCAGAGCCCGAAGAGCCCGCAGCAGCGTCTCCTGGAGCTGCTCCACCGAAGCGGAATTCTCCATTCCCGAGCGGTCTGTGGGGAAGACGACAGCAGTGAG
SAO     (754)  TGAGGGCCGATTCTTCAGGACCAGGGCCCGAAGAGCCCGGAGCAGCGTCTCCTGCAGCTGTTCTACAGAGGCCGAGTTCTCCATACCTGATCGATCTGTTGGGCAAAAGGAAAAGGGCAA
NAO     (758)  TGAGGGCCGATTCTTCAGGACCAGCGCCCGAAGCGCCCGGAGCAGGGTCTCCTGCAGCTGTTCCACAGAGGCCGAGTTCTCCATACCGGATCGATCTATTGGGTGAAGGGAAAAGGGCAA
Potoro  (773)  TGAGGGCCTGTTCTTTAGGACCAGTGCCCGAAGTGCTCGGAGCAGTGTCTCCTGCAACTGTTCCACAGAGGCTGAGTTCTCCATACCTGATCGGTCTGTTGGGAGAAGGGAAGAGGACAG
Wallaby (776)  TGAGGGCCTGTTCTTTAAGACCAGTGCCCGAAGTGCTCGGAGCAGTGTCTCCTGCAACTGTTCCACAGAGGCCGAGTTCTCCATACCTGATCGGTCTGTTGGGAGAAGGGAAGAAGACAG

                                                                                   TRα2 stop
Rat     (904)  GCAAGCTCCCTG-----AGCTCCTCTTCCTCTGACGAGGACACGGAGGTCTGCGAGGACCTGGCAGGCAAGGCAGCCTCTCCCTGAGGCCCCTTGGAGGGCGATGGGGAAGGAGAAGGAG
SAO     (874)  GAAGTGACC-AA-----AGGTG--TCAGGCTGGAAAGGTCCCTGGACATAAGGGCTACATTGTTCACCAGAAA-----TCTAGGAGGGAGCTGACATAATGGATAGGATGCCAGGCCTG
NAO     (878)  GAAGTGACTGAG------GGGTG--TCAGGCTGGAAAGGTCCCTGAACCAGAAATCTA----GG--A---GAGA-----GCTAGGTGG---CGGAG----TGGATGGAATGTCAGGTCTG
Potoro  (893)  GAAGTGACTGAG-----AGGGGG--TCAGGCTGGAAAGCTCCCTAGACATGGGCACTACATTGTTCACAAGAAA-----TCCAGGATG---CTG-------GAAGAGGATGTGAAA---G
Wallaby (896)  GGAGTGACTGAGGGGAGAGGGGGGCTCAAGCTGGAAAGCTCCCTGGACATGGGCATTACGTTGTTCAAAAGAAA-----TCCAGGATG---CTG-------GGAGAGGACGTGAAA---G

Rat     (1019) CATGCTACTCCTTCTCCAGGGCCTCTGCCCTAGAGCTGGGCACGCCCACATGCTGGGAGCAAGGCCACAGGC---TGGCCGCCTCCCACTCAAGACCACCCTCCTACCCCTGAGCACGCC
SAO     (981)  GAGTCTAGGCGA----CCTGGGTTCTAATCTGGTCTTACATACTTCCTAGGTATGTGACCCCTGGGCAAGGCACTTAACCCTGTTTGCCTAGCCCTTACCCTTCCAACTTATAGTTGATA
NAO     (969)  GAGTCTAGACAA----CCTGGGTTCTAATCTGGTCTCGAATACATCCTAGCTATGTGACCCCGGGGCAAGTCACTTAATCCTGTTTGCCTAGCCCTTACCATTCCATCTTATAGTTGATA
Potoro  (988)  GACACTACAGGA----C----------------------ACACTGCCT-----------CTAGGGGCCAAG------------------------------------------------
Wallaby (998)  GACACTAAAGGA----C----------------------ACACTGCCT-----------CTAGGGGCCAGG------------------------------------------------

Rat     (1136) ACACGTGGCCAAGCTCCCTTGTTATTTGTTCTGTAGTTCCCTCTGCCTGGGATGCCCTTCCCCCTCTCTATGCCTGGCAATACCTTGTCCCTTTGAGGCCCCACTCAAGTGTCACCTCCT
SAO     (1097) CCAAGACAGAAAGGAAGGATTAAAAAGAAGAAATCCAGGACAATGAAAGGATGGGAC-----------ACAAGACACAGACTCTTGCTGCCTGTAGGGTGCGGGAAAG------CAGCA
NAO     (1085) CGAAGCCAGAAAGGAAGGATTAAAAAAAAGAA-----GAAGAAGAAATCCAGGATAT-----------GGAGAGGACAGGACACTACAAGACACACGGAC---TCTCG------CTGCC
Potoro  (1022) ---------GAAGGGAGCATCA---------------------TGAATG----------------------------CAGA---------------GGTCCTATCAAG------CA---
Wallaby (1032) ---------AAAGGGAGCATCA---------------------TGAGTG----------------------------CAGAA--------------GGTCCTATCAAG------CA---

Rat     (1256) TCCCCAGCTCTCCCAGGCAGAAATAGTTGTCTGTGCTTACGTGGTTCATGCTTCTTCTGTGACACTTCTCGCTGTTTTATAATTAGTCGGGCATGAGTCTGTTTCCCAAGCTAGACTGTG
SAO     (1199) TCTC-GAGTCTTCAGAGGATA----------CCA-CAGAGGACACAGAAGCTGTTTCC-TGGCA--TCTC----TTTGCATATAGGATAGGTGGGGCTGTAGAATACCAGCCTGCAAATG
NAO     (1179) TTTCGGATGAGGAAAAGGA------------GCATCTCAAGTCACAGAAGCTGTTTCC-TGGCA--TCTC----TTTGCATGTGGGAAAGG-GGGGGTATACAATACCAGCCTGCAAATG
Potoro  (1059) TCTC-AAGTCTTCAGAGGACA----------TGAACAGAGGGTGCAGAAGCTTATTTC-TGCCA--TCCC----TTT---------------------------------------AGATG
Wallaby (1070) TCTCCAAGTCTTCAGAGGACA----------GGAGCAGAGGGTACAGAAGCTTATTCC-TGCCA--TCCC----TTT---------------------------------------ACATG

                                                                    TRα2 poly(A) site
Rat     (1376) TCTGAATCATGTCTGTAGCCCAGTGCCCAGTGCCGGGCCTGGCATAGAGTAGGAACTCCATAAAAGGTATGTTGAATTGAACTGCGTGTGCCTCCTC-CAGGGTCAGGTGAGAGCTTGGC
SAO     (1300) TAGGAGGGAA--CTGA----------CCAGGGACTGAGA-GACCGAGAGCCTGCAAAC------ATGGCTGGCCCA--ACATGCCCTCCCCTTCCCCTGCGGCTAGGGC-----CATGAC
NAO     (1279) CAGGAGGGAA--CTAA----------CCAG-GACTGAAA-AACCAAGAGCCTGCAA-C------GTGGCTGGCCCA--GCATCCCCTCCCCTTTCCCTGTGGCTAAGGC-----CATGGC
Potoro  (1123) CAGGAGAGAT--CTGA----------CTAGAGACGGGGA-GGCCAAGAGCCGGTAG-C------ACGGCTGTTACA--GCATCCCCTCCACATCCCCTCTGGTTGAGGTG---GCATAAC
Wallaby (1135) CAGAAGAGAT--CTGA----------CTAGAGACAGGGA-GGCCAAGAGCTGGCAG-C------ATGGCTGTTACA--GCATCCCCTCCACATCCCCTCTGGCTGGGGTG---GCATAGC

                   Rev-erbα 5'splice site
Rat     (1495) TTACCTGCAGAGACAAGTACCACTGCCGTGAAAAGGCCCAGCTCCTCCTCAGTAAGCGCCAGAGAGTTGAGCTTCTCGCTGAAGTCAAACATGGCATTGAGCAGGTCACCCATGCCCATG
SAO     (1394) CTACCTGCAGAGACAAGCACCACTGCAGTAAACAGTCCCAGCTCCTCTTCTGTGAGGGCCAAGGAGTTAAGCTTTTCACTGAAGTCGAACATGGCATTGAGCAGGTCACCCATGCCCATG
NAO     (1371) CTACCTGCGGAGACAAGCACCACTGCAGTAAACAGACCCAGCTCCTCTTCCGTGAGGGCCAAGGAGTTAAGCTTTTCACTGAAGTCGAACATGGCATTGAGCAGGTCACCCATGCCCATG
Potoro  (1218) CTACCTGCAGAGACCAGCACTACTGCAGTAAACAGCCCCAGCTCCTCTTCTGTGAGGGCCAGGGAGTTCAGCTTTTCACTGAAGTCGAACATGGCATTGAGCAGGTCCCCCATCCCCATA
Wallaby (1230) CTACCTGCAGAGACCAGCACTACTGCAGTGAACAGCCCCAGTTCCTCTTCTGTGAGGGCCAGGGAGTTCAGCTTTTCACTGAAGTCGAACATGGCATTGAGCAGGTCCCCCATCCCCATA
```
