# Supplementary figures and images for: Structure and expression of two nuclear receptor genes in marsupials: insights into the evolution of the antisense overlap between the α-thyroid hormone receptor and Rev-erbα
Source: BMC Mol Biol. 2010 Dec 10;11:97. doi: 10.1186/1471-2199-11-97 (PMC3047299; doi:10.1186/1471-2199-11-97)

Additional figure 3

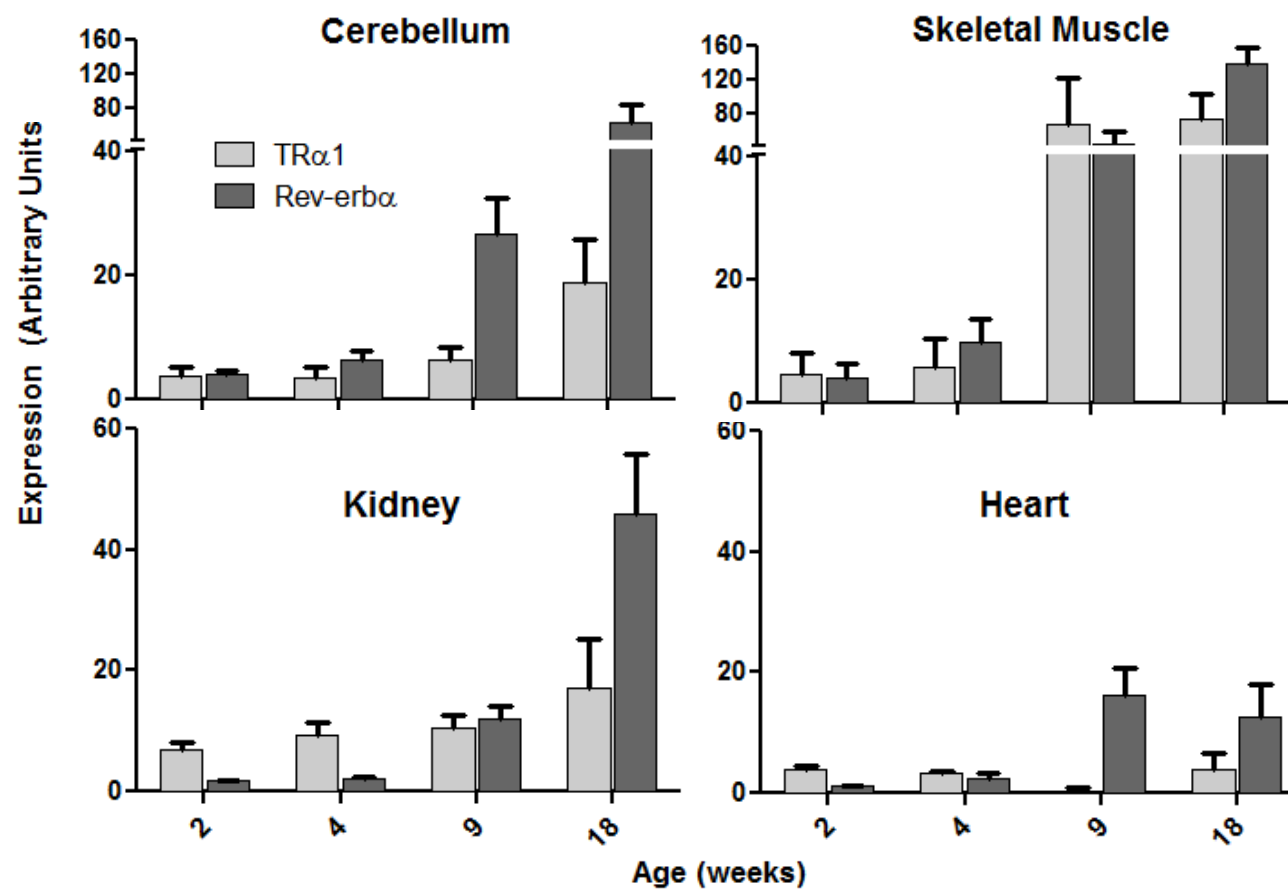

Supplement: Additional file 3 — Expression of TRα1 and Rev-erbα in cerebellum, kidney, skeletal muscle and heart expression isolated from 2 to 18 week old opossums. Tissues from 2, 4 9 and 18 week old opossums (M. domestica) were assayed for TRα1 and Rev-erbα expression as in Figure 3A. Results are averages of three animals, with each assay performed in triplicate. Brackets indicate standard deviations. [file 1471-2199-11-97-S3.PDF]
