# Supplementary material for: Structure and expression of two nuclear receptor genes in marsupials: insights into the evolution of the antisense overlap between the α-thyroid hormone receptor and Rev-erbα
Source: BMC Mol Biol. 2010 Dec 10;11:97. doi: 10.1186/1471-2199-11-97 (PMC3047299; doi:10.1186/1471-2199-11-97)
Supplement: Additional file 4 — Table showing realtime RT-PCR data illustrating absence of detectable TRα2 mRNA in opossum tissues. Uncorrected threshold values (CT values) are shown for TRα2 mRNA in upper left of the box for each age and tissue. For comparison, values for TRα1 in parallel runs are shown the lower right of each box. Each value is the average of 3 replica determinations from a single run, with samples failing to reach the threshold arbitrarily set to 42 in determining the average. Boxes outlined in bold show runs where measurements for TRα1 and TRα2 were carried out simultaneously. All samples were assayed for TRα2 with two different primer sets. Values shown without asterisks were measured with the primer pair evaluated in Figure 3E. Asterisks indicate measurements with an alternate TRα2 primer pair also used in Figure 3D. Replica values for TRα1 show little variation (SD< 0.2 for all but 4 measurements). CT values for TRα2 typically showed high values ( > 38) and/or substantial variation (SD > 1) characteristic of non-specific products over a 44 cycle run. [file 1471-2199-11-97-S4.PDF]

# Additional file 4 (Table 1)

| Age | Head | Body | Cerebell. | Kidney | Heart | Muscle | Testes |
|---|---|---|---|---|---|---|---|
| Day 0 | 38.86* / 20.62 | 40.84* / 20.27 | | | | | |
| Week 1 | 38.94* / 20.14 | 38.18* / 20.77 | | | | | |
| | C. Cortex | Liver | | | | | |
| Week 2 | 36.69 / 21.62 | 37.35 / 21.35 | 40.66 / 21.75 | 36.58 / 21.50 | 35.17 / 20.55 | 37.48 / 20.68 | |
| Week 4 | 39.23* / 24.72 | 41.01 / 21.92 | 41.63* / 24.05 | 35.15 / 21.05 | 34.85 / 20.69 | 37.48 / 25.82 | |
| Week 9 | 38.51 / 25.15 | 37.03 / 22.35 | 38.42 / 24.33 | 36.33 / 20.81 | 42.00 / 24.00 | 40.23 / 21.08 | 37.43 / 22.04 |
| Week 18 | 39.23 / 20.89 | 40.21 / 21.59 | 39.43 / 21.27 | 41.58 / 20.43 | 39.93 / 20.61 | 40.26 / 20.65 | 39.13 / 22.69 |
